# Loss of C9orf72 Enhances Autophagic Activity via Deregulated mTOR and TFEB Signaling

**DOI:** 10.1371/journal.pgen.1006443

**Published:** 2016-11-22

**Authors:** Janet Ugolino, Yon Ju Ji, Karen Conchina, Justin Chu, Raja Sekhar Nirujogi, Akhilesh Pandey, Nathan R. Brady, Anne Hamacher-Brady, Jiou Wang

**Affiliations:** 1 Department of Biochemistry and Molecular Biology, Bloomberg School of Public Health, and Department of Neuroscience, School of Medicine, Johns Hopkins University, Baltimore, Maryland, United States of America; 2 McKusick-Nathans Institute of Genetic Medicine, Johns Hopkins University, School of Medicine, Baltimore, Maryland, United States of America; 3 Department of Molecular Microbiology and Immunology, Bloomberg School of Public Health, Johns Hopkins University, Maryland, United States of America; The Jackson Laboratory, UNITED STATES

## Abstract

The most common cause of the neurodegenerative diseases amyotrophic lateral sclerosis and frontotemporal dementia is a hexanucleotide repeat expansion in *C9orf72*. Here we report a study of the C9orf72 protein by examining the consequences of loss of C9orf72 functions. Deletion of one or both alleles of the *C9orf72* gene in mice causes age-dependent lethality phenotypes. We demonstrate that C9orf72 regulates nutrient sensing as the loss of C9orf72 decreases phosphorylation of the mTOR substrate S6K1. The transcription factor EB (TFEB), a master regulator of lysosomal and autophagy genes, which is negatively regulated by mTOR, is substantially up-regulated in C9orf72 loss-of-function animal and cellular models. Consistent with reduced mTOR activity and increased TFEB levels, loss of C9orf72 enhances autophagic flux, suggesting that C9orf72 is a negative regulator of autophagy. We identified a protein complex consisting of C9orf72 and SMCR8, both of which are homologous to DENN-like proteins. The depletion of C9orf72 or SMCR8 leads to significant down-regulation of each other’s protein level. Loss of SMCR8 alters mTOR signaling and autophagy. These results demonstrate that the C9orf72-SMCR8 protein complex functions in the regulation of metabolism and provide evidence that loss of C9orf72 function may contribute to the pathogenesis of relevant diseases.

## Author Summary

*C9orf72* is one of many uncharacterized genes in the human genome. The presence of repeated nucleotides in the non-coding region of the *C9orf72* gene (GGGGCC) has been linked to the neurodegenerative diseases Amyotrophic Lateral Sclerosis (ALS) and Frontotemporal dementia (FTD). However, how the presence of these repeats in the gene leads to neurodegeneration is unknown. One possible explanation is that the repeats lead to a reduced expression of the *C9orf72* gene and loss of function of the C9orf72 protein. Although *C9orf72* is well-conserved among multi-cellular organisms, its protein function remains to be determined. In this study, we demonstrated that loss of C9orf72 reduces mTOR signaling and enhances autophagy. mTOR signaling and autophagy are important for the cellular maintenance of metabolic balances, especially under stress conditions. C9orf72 protein exists in a complex with another DENN-like protein, SMCR8, which also regulates mTOR signaling and autophagy. We generated mice lacking C9orf72, which died prematurely and showed dramatic upregulation of TFEB, a crucial transcriptional regulator of autophagy and lysosomal genes, that integrates mTOR activity state and autophagic capacity. We propose that C9orf72 function is important for metabolic control and its deficiency can contribute to the development of neurodegenerative diseases.

## Introduction

Amyotrophic lateral sclerosis (ALS) is a fatal neurodegenerative disease characterized by the progressive degeneration of motor neurons. Frontotemporal dementia (FTD) is the second most common type of dementia in people younger than 65 and is characterized by degeneration of the frontal and temporal lobes of the brain. A hexanucleotide repeat expansion (HRE), (GGGGCC)n, in the promoter or intron of the uncharacterized gene, chromosome 9 open reading frame 72 (*C9orf72*), has been found to be the most common cause of both ALS and FTD [1, 2] and has been linked to a number of other neurological disorders. How the *C9orf72* HRE leads to neurodegeneration remains to be determined, although both gain-of-toxicity and loss-of-function mechanisms have been proposed. The gain-of-toxicity mechanisms involve both RNA and protein products generated from the expanded hexanucleotide repeats. For example, RNAs containing the expanded repeats can interfere with the functions of specific RNA-binding proteins [3–5], and toxic repeat polypeptides can be generated through repeat-associated non-ATG-dependent translation [6–10]. However, the HRE could be pathogenic through loss-of-function mechanisms when the expression of the *C9orf72* gene is disrupted. Multiple studies have demonstrated that *C9orf72* RNA and protein levels are reduced in patient cells and brains [11–15]. Although partial knockdown of C9orf72 in the brain or its neural-specific deletion does not affect survival in mice [16, 17], loss of *C9orf72* orthologs in zebrafish and *C*. *elegans* has deleterious effects [18, 19].

Studies of these loss-of-function mechanisms are hampered by a lack of knowledge about the physiological function of the C9orf72 protein. Bioinformatic analysis suggested that C9orf72 is a DENN-like protein [20, 21], which is a family of proteins that regulate small GTPases and membrane trafficking. DENN domain-containing proteins have also been implicated in autophagy and in the mammalian target of rapamycin (mTOR) signaling pathways [22]. Although a recent study has reported that C9orf72 regulates autophagy and endosomal trafficking [23], the function of the C9orf72 protein remains largely unknown.

Here we report the findings in mice and human cells that loss of C9orf72 inhibits mTOR signaling and leads to a profound upregulation of transcription factor EB (TFEB) and enhanced autophagy flux. We further show that C9orf72 interacts with another DENN-like protein Smith-Magenis syndrome chromosome region candidate 8 (SMCR8), which also regulates mTOR signaling and autophagy. The results suggest that a deficiency in the function of C9orf72 may contribute to the pathogenesis of relevant neurodegenerative diseases.

## Results

### C9orf72 KO mice show decreased life span

**Fig 1:**
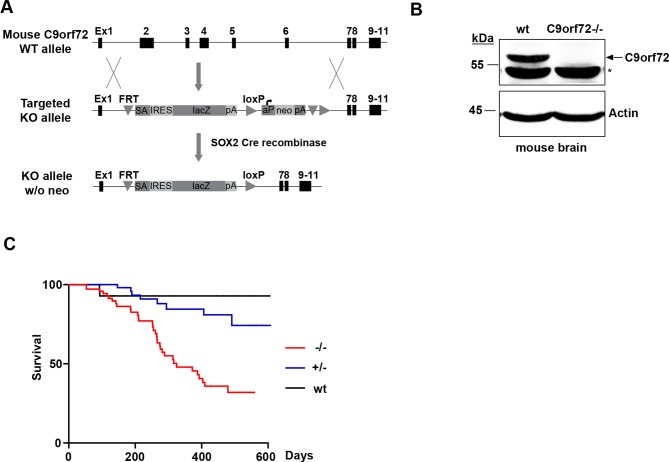
C9orf72 knockout mice show age-dependent lethality. **A)** Schematic representation of the generation of the C9orf72 KO mice. Diagram depicts the mouse C9orf72 wild-type allele, C9orf72 targeting cassette, and the KO allele after SOX-Cre recombination. **B)** Immunoblot analysis of C9orf72 -/- and wild-type littermate brain homogenates. C9orf72 antibody detects a band at 55 kDa not present in C9orf72-/- lysates. **C)** Survival analysis of C9orf72-/-, C9orf72+/-, and wild-type mice. Lifespans were monitored and plotted using Kaplan-Meyer curve. This analysis revealed a loss of C9orf72 causes a significant decrease in survival when compared with wild-type littermates (n = 74, C9orf72-/-; n = 79, C9orf72+/-; n = 31, wild-type, *p<0.005).

To study the physiological functions of C9orf72 in mammals, we generated a knockout (KO) mouse model lacking the protein. Human *C9orf72* has one orthologous gene in the mouse, 3110043O21Rik, which is located on chromosome 4. For convenience, we refer to the mouse gene as *C9orf72* hereafter. The mouse *C9orf72* gene is predicted to produce seven transcripts, three of which are protein-coding, as compared to the human *C9orf72* gene, which produces three transcripts and two protein isoforms. The mouse C9orf72 proteins share 98% identity with their human C9orf72 counterparts (S1 Fig). We generated C9orf72 KO mice by using a mouse embryonic stem (ES) cell line that contains a heterozygous allele of a 7754 base pair deletion in the *C9orf72* gene. This deletion results in the removal of exons 2–6 and is predicted to produce nonfunctional truncated protein products from all three protein-coding transcripts of the mouse *C9orf72* gene (Fig 1A). We further removed the neomycin cassette by crossing the C9orf72 KO male mice carrying the original targeted allele with SOX2-Cre transgenic females (Fig 1A). Western blotting of brain homogenates from C9orf72 wild-type and KO littermates, using an antibody predicted to detect all mouse C9orf72 isoforms, showed a protein band at 55 kDa (corresponding to mouse isoform 1), not present in the C9orf72-/- samples (Fig 1B), confirming that our KO mice lack C9orf72 in brain. We were unable to detect the other two mouse C9orf72 isoforms, suggesting that mouse isoform 1 is the major isoform in the mouse brain. The homozygous C9orf72 KO mice showed a decrease in survival compared with littermates, with more than 50% dead in 600 days (Fig 1C). This decrease in survival was also observed in heterozygous C9orf72+/- animals to a lesser degree with only about 20% dead in 600 days. Both C9orf72 homozygous and heterozygous knockout mice developed normally before exhibiting rapidly progressive lethargy before death. The stage of lethargy could last for days up to a month. At the end stage, the animals showed a lack of excitability or response to external stimuli (S1 Movie). In post-mortem examination, consistent with recent reports of immune dysregulation in C9orf72 knockout mice [24–27], we observed splenomegaly in the C9orf72-/- mice. The spleen was generally increased in length from ~3/4 inches to 1–1.25 inches. In addition, we frequently observed potential tumors in the thymus or in the regions of the abdomen. There was no obvious neuronal cell death in brain or spinal cord, but functional deficits of the nervous system could not be excluded. The exact cause of death for these C9orf72 knockout mice remains to be determined.

### Loss of C9orf72 impairs mTOR activation

**Fig 2:**
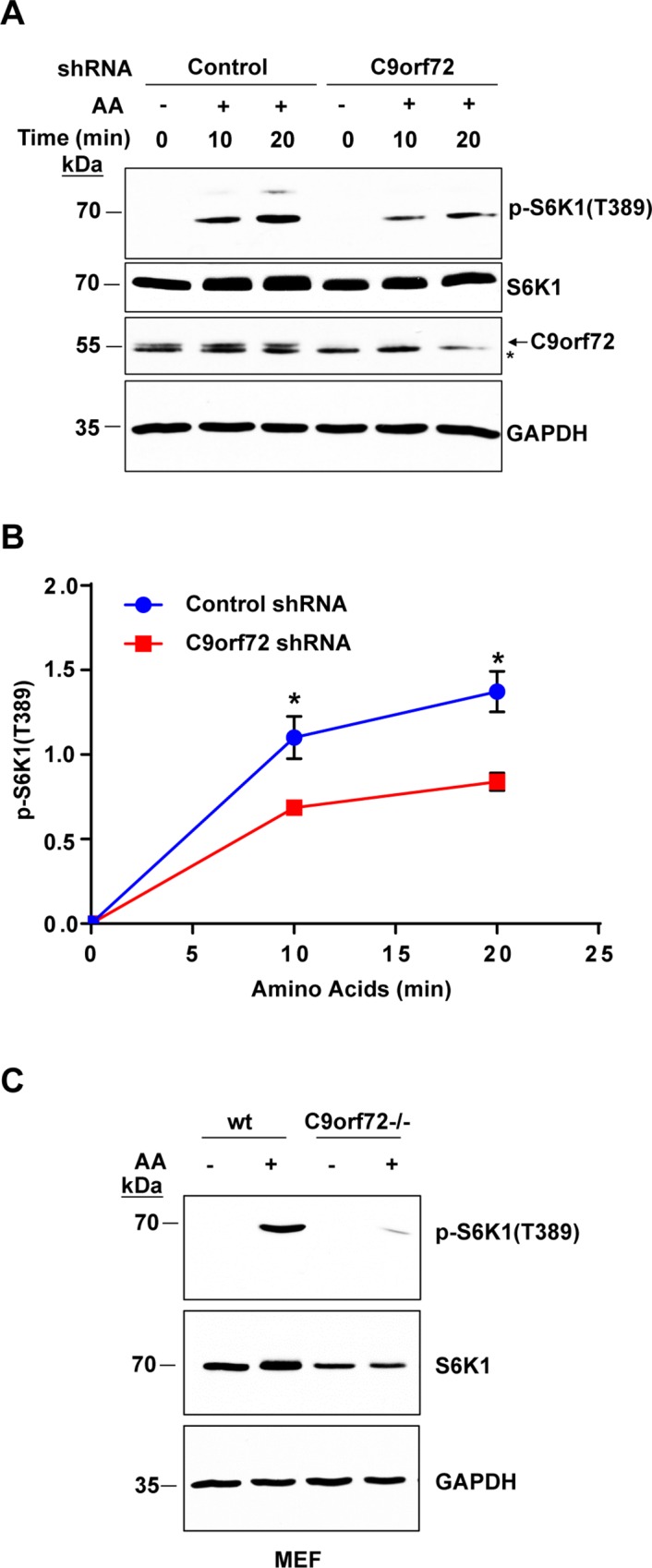
Loss of C9orf72 decreases mTOR activation. **A)** Immunoblot analysis of mTOR activity after starvation and amino acid stimulation. HEK293T cells were transfected with control or C9orf72 shRNA for 72 hours before treatment. Cells were starved for 50 min before being supplemented with amino acids for 10–20 min prior to lysate collection. The mTOR activity was assessed by immunoblotting for the phosphorylation of its downstream substrate S6K1. Arrow points to C9orf72 and asterisk indicates a cross-reacting band. **B)** Quantification of p-S6K1 (T389) levels after starvation and amino acid stimulation in control and C9orf72 knockdown cells from three independent experiments. Knockdown of C9orf72 significantly decreased p-S6K1 levels compared with cells treated with control shRNA (n = 3, *p<0.5). **C)** Immunoblot analysis of mTOR activity in C9orf72-/- MEFs after starvation and amino acid stimulation. C9orf72-/- and wild-type cells were starved for 50 min using EBSS and supplemented with amino acids for 10 min prior to lysate collection. C9orf72-/- MEF cells show a decrease in p-S6K1 when compared with wild-type cells. Student’s *t* test is used and data is presented as mean ± SEM.

Although we observed no obvious neuronal defects in C9orf72 KO mice, it is possible that C9orf72 has functions in the nervous system in response to stresses. Thus, we asked if mTOR signaling is altered when C9orf72 is absent, since mTOR signaling is a central signaling pathway that senses the stresses related to nutrient availability, oxygen, and energy levels [28]. Also, DENN-like proteins have been implicated in mTOR signaling and nutrient sensing [29–31] and C9orf72 contains DENN domains. We monitored mTOR activity by assessing the phosphorylation of its downstream target ribosomal protein S6 kinase B1 (S6K1). Cells were starved for amino acids for 50 minutes before amino acids were added back to induce the phosphorylation of S6K1. Interestingly, knockdown of C9orf72 in HEK293T cells resulted in a decrease in the phosphorylation of S6K1 within 10 to 20 minutes after addition of amino acids, as compared with control cells transfected with scrambled control shRNAs (Fig 2A and 2B). These results suggest that the loss of C9orf72 decreases mTOR activation after amino acid stimulation. To study the molecular defect in the complete absence of C9orf72 protein, we generated mouse embryonic fibroblasts (MEFs) from C9orf72 wild-type and KO littermates. And we assessed the phosphorylation of S6K1 in the C9orf72-/- MEF lines. Phosphorylation of S6K1 was decreased in C9orf72-/- MEF lines compared with lines derived from wild-type littermates (Fig 2C), suggesting that mTOR activation after amino acid stimulation is diminished in the absence of C9orf72.

**Fig 3:**
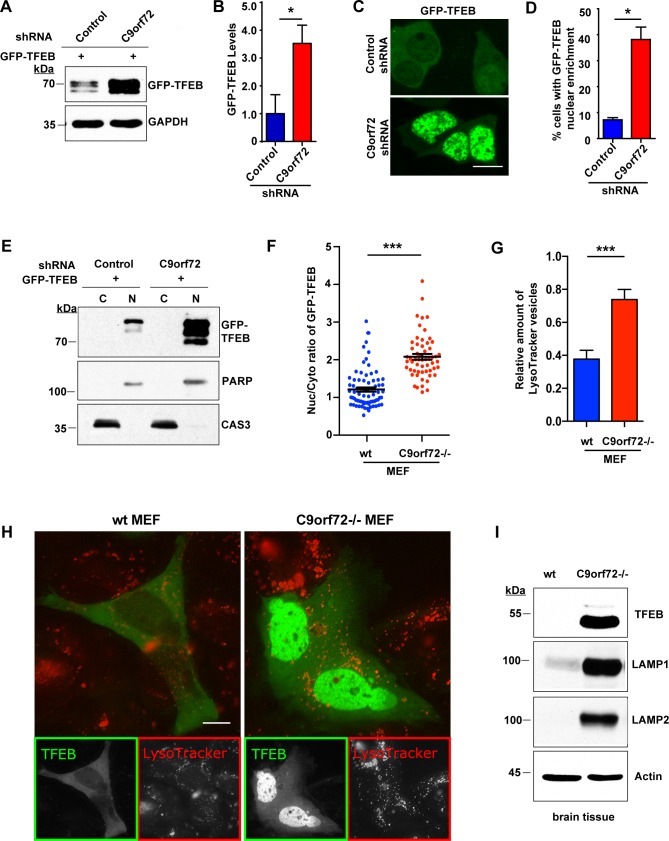
Loss of C9orf72 increases TFEB in the nucleus. **A)** Western analysis of TFEB levels after knockdown of C9orf72 in HEK293T cells. HEK293T were transfected with GFP-tagged TFEB and C9orf72 shRNA or control shRNA and then the lysates were analyzed using an antibody to GFP. **B)** Quantification of GFP-TFEB levels after knockdown of C9orf72 in HEK293T cells. Loss of C9orf72 significantly increases GFP-TFEB protein levels (n = 3, *p<0.05). **C)** Representative image of the nuclear enrichment of GFP-TFEB after knockdown of C9orf72 in HEK293T cells. **D)** Quantification of GFP-TFEB localization. Loss of C9orf72 increases the number of cells that show strong nuclear levels of GFP-TFEB (n = 4 control groups of 277 cells and n = 3 groups of 384 cells for C9orf72 in two independent trials, *p<0.05). **E)** Western blot analysis of nuclear fractionation of GFP-TFEB in HEK293T cells. Equal amounts of proteins from nuclear and cytoplasmic fractions (a ratio of approximately 6:1 in nuclear to cytoplasmic parts) were loaded. The GFP-TFEB levels were significantly increased in the nucleus upon C9orf72 knockdown. PARP and Caspase-3 were used as the loading control for the nuclear and cytoplasmic fractions, respectively. **F)** Quantification of nucleocytoplasmic distribution of GFP-TFEB fluorescence in C9orf72-/- and wild-type MEF cells (n = 74 for control and n = 57 for C9orf72-/- in four independent trials, ***p<0.0001). **G)** Quantification of LysoTracker stained vesicles in in C9orf72-/- and wild-type MEF cells (n = 40 for control and n = 64 for C9orf72-/- in three independent trials, ***p<0.0001). **H)** Representative images of the nuclear enrichment of GFP-TFEB (green) and the increased LysoTracker vesicles (red) in C9orf72-/- MEF cells. **I)** Analysis of the levels of TFEB and its downstream targets in C9orf72 KO mice. Immunoblot analysis of C9orf72-/- mouse and wild-type littermate brain homogenates shows a dramatic increase of TFEB in C9orf72 KO animals compared with wild-type control animals. TFEB targets LAMP1 and LAMP2 were also increased in the KO mice. Scale bars: 10 μm. Student’s *t* test is used and data is presented as mean ± SEM.

Subsequently, we asked whether the observed reduction of mTOR activation in the absence of C9orf72 impacts the function of TFEB, a transcription factor that is a master regulator of lysosome biogenesis and autophagy-related genes, and a substrate of mTOR [32]. In an autoregulatory loop, nuclear translocation of TFEB leads to increased expression of itself. Phosphorylation of TFEB by mTOR prevents its translocation to the nucleus and causes down-regulation of TFEB. We transfected GFP-TFEB into HEK293T cells and observed that knockdown of C9orf72 resulted in a significant increase in GFP-TFEB levels (Fig 3A and 3B), consistent with the decrease in mTOR activity. Moreover, imaging analysis indicated that nuclear localization of GFP-TFEB was significantly increased upon knockdown of C9orf72 as compared with cells treated with control shRNAs (Fig 3C and 3D). Western blotting of the nuclear and cytoplasmic fractions further confirmed that GFP-TFEB was enriched in the nucleus upon knockdown of C9orf72 (Fig 3E). Next, we validated these results in C9orf72-/- and wild-type MEF cells. Consistently, the complete absence of C9orf72 led to a significant increase in the nucleus to cytoplasm ratio of GFP-TFEB signals (Fig 3H and 3F). Furthermore, consistent with the notion that TFEB promotes the biogenesis and activity of lysosomes [32], we observed a significant increase in the number of LysoTracker-stained acidic vesicles in the C9orf72-/- MEF cells, confirming functional consequences on lysosomes of enhanced nuclear TFEB (Fig 3H and 3G).

We then questioned whether our results held true *in vivo*. Analysis of brain homogenates by western blotting from all examined C9orf72 KO mice showed a dramatic increase in endogenous TFEB levels compared with wild-type controls (Fig 3I), consistent with our results in tissue culture. We next asked whether downstream targets of TFEB were also increased by loss of C9orf72. Indeed, western blot analysis of lysosome-associated membrane glycoprotein 1 (LAMP1), which is a transcriptional target of TFEB [33], indicated that LAMP1 was profoundly increased in the C9orf72 KO mouse brains (Fig 3I). Another related lysosomal protein LAMP2 was also markedly increased in the absence of C9orf72 in the KO mouse brains. Taken together, these results suggest that, consistent with the inhibition of mTOR signaling, loss of C9orf72 increases TFEB activity.

### Loss of C9orf72 increases autophagic flux

Since we observed a function of C9orf72 in mTOR signaling and mTOR is known to negatively regulate autophagy, we assessed the levels of the autophagy marker LC3 by immunoblotting in these cells. During autophagy, LC3I is processed to LC3II via lipidation, which allows for insertion of the LC3 protein into the autophagosome membrane. Our results show a significant increase of LC3I in C9orf72-/- MEFs when compared with wild-type MEFs, indicating that basal autophagy is altered in these cells (S2A and S2B Fig). To test the role of C9orf72 in neurally differentiated cells, we generated embryonic stem cells from C9orf72 KO mice and littermate controls and differentiated them into the motor neuron precursors that further grew into mature motor neurons (~40% of the culture) plus astrocytes and oligodendrocytes. We assessed the level of LC3 by immunoblotting and found that the C9orf72-/- cells enriched with motor neurons showed a substantial accumulation in LC3I (S2C Fig), in line with what was observed in C9orf72-/- MEFs.

**Fig 4:**
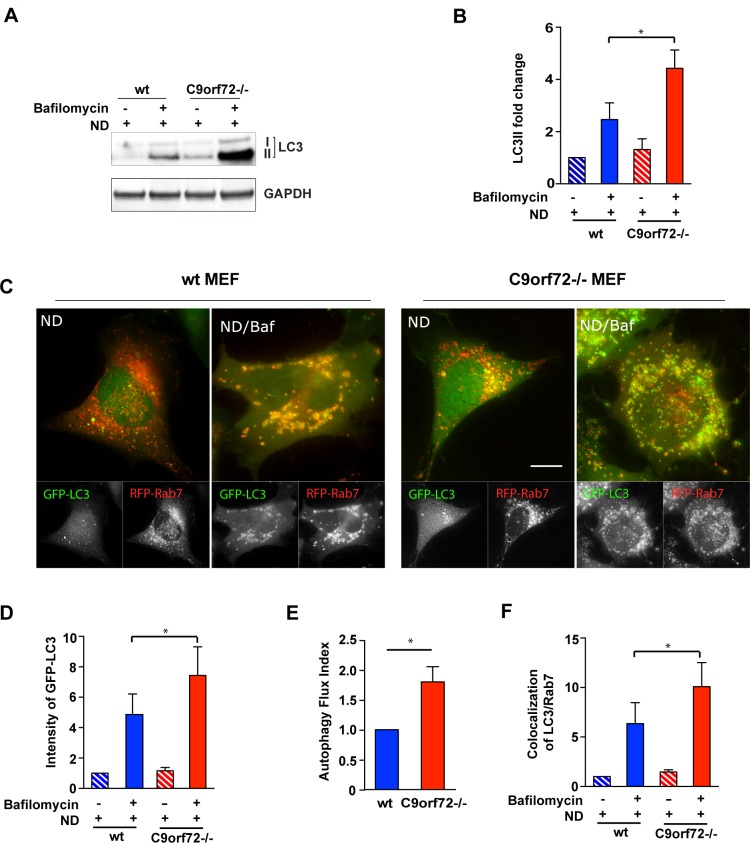
Loss of C9orf72 increases starvation-induced autophagy flux. **A)** Representative image of western blot analysis of LC3 in nutrient deprivation (ND) conditions. Wild-type or C9orf72-/- MEF cells were treated with or without Bafilomycin in nutrient deprivation conditions for three hours, and the LC3I and LC3II levels were determined by western blotting. **B)** Quantification of western blot analysis of LC3II as in A). LC3II level was significantly increased in C9orf72-/- cells treated with Bafilomycin compared with wild-type cells (n = 3, *p<0.05). **C)** Representative live cell images of RFP-Rab7/GFP-LC3 co-localization in C9orf72-/- MEFs. RFP-Rab7 and GFP-LC3 were transfected in wild-type or C9orf72-/- cells and treated with Bafilomycin in nutrient deprivation conditions. **D)** Quantification of GFP-LC3 intensity in C) (n = 3 independent trials with a total of 125 cells, *p<0.05). **E)** Quantification of an autophagy flux index in C) defined as the difference in the volumes of LC3-positive vesicles before and after Bafilomycin treatment (n = 3 independent trials with a total of 125 cells, *p<0.05). **F)** Quantification of the fraction of LC3/Rab7 colocalizing vesicles in C) (n = 3 independent trials with a total of 125 cells, *p<0.05). Scale bar: 10 μm. Student’s *t* test is used and data is presented as mean ± SEM.

The decreases in LC3II/LC3I ratio observed in our western blots can indicate a defect in lipidation or an increase in degradation via the lysosome. To distinguish between these two possibilities, we assessed LC3 levels after nutrient deprivation-induced autophagy in the absence and presence of the lysosomal inhibitor Bafilomycin in C9orf72-/- and wild-type MEF cells. We found that the Bafilomycin-induced accumulation of LC3II was significantly enhanced in C9orf72-/- MEFs compared with wild-type MEFs (Fig 4A and 4B), indicative of an enhanced autophagic flux in C9orf72-depleted cells. To further examine the status of autophagic flux, we analyzed the numbers of LC3-positive autophagic vesicles and the colocalization between LC3 vesicles and Rab7, a late endosome-/lysosome-associated GTPase that marks mature autophagolysosomes [34–36] (Fig 4C–4F). Quantification of LC3-positive vesicles in the absence and presence of Bafilomycin, demonstrates that, consistent with western blot results, the number of LC3-positive autophagic vesicles was significantly increased in C9orf72-/- MEFs (Fig 4D). An autophagic flux index, defined as the difference in the volumes of LC3-positive vesicles before and after Bafilomycin treatment, was quantified, further confirming the increased autophagic flux capacity in C9orf72-/- MEFs (Fig 4E). Similarly, we observed an increase in the colocalization of LC3-positive autophagic vesicles with Rab7-positive vesicles (Fig 4F), confirming enhanced autophagolysosome formation. In addition to induced autophagy, we also examined basal autophagic flux under fully supplemented nutrient conditions in the absence of C9orf72 (S3 Fig). Despite of relatively low level of signals, the LC3/Rab7 vesicle colocalization assay indicated a trend that there were more LC3-positive vesicles and more colocalized LC3/Rab7 vesicles in Bafilomycin-treated C9orf72-/- MEFs than in wild-type control cells (S3A Fig). This result is consistent with the western analysis of LC3 protein levels, in which LC3II accumulated robustly in Bafilomycin-treated C9orf72-/- MEFs (S3B Fig).

In addition to MEF cells, we observed a similar result in HEK293T cells for autophagic flux after knockdown of C9orf72. Under nutrient deprivation, in cells treated with C9orf72 shRNA, despite a decrease in LC3II/LC3I ratio before Bafilomycin treatment, lysosomal inhibition induced a robust accumulation of LC3II (S3C Fig), suggesting that the total autophagic flux was increased. Taken together, the observed increases in autophagic activity are consistent with the impairment of mTOR signaling and the profound increase of TFEB as results of loss of C9orf72.

Of note, our results do not rule out the possibility that C9orf72 functions in other aspects of autophagy. For example, we examined the activity of ATG4B, which catalyzes the cleavage of proLC3 to produce LC3I and also removes LC3II from the autophagosome membrane after it fuses with the lysosome (S4A Fig). Knockdown of C9orf72 in HEK293T cells resulted in a significant decrease in the signal of an ATG4B activity luciferase reporter when compared with control cells (S4B Fig), suggesting that ATG4B activity is impaired under basal conditions. However, no change was detected in ATG4B protein levels by western blotting upon knockdown of C9orf72 (S4C Fig and S4D Fig), indicating that the reduction in ATG4B activity was not due to a decrease in its protein level. The unchanged level of ATG4B protein could presumably make it readily available to support the enhanced autophagic flux observed in nutrient deprivation-treated C9orf72 deficient cells.

**Fig 5:**
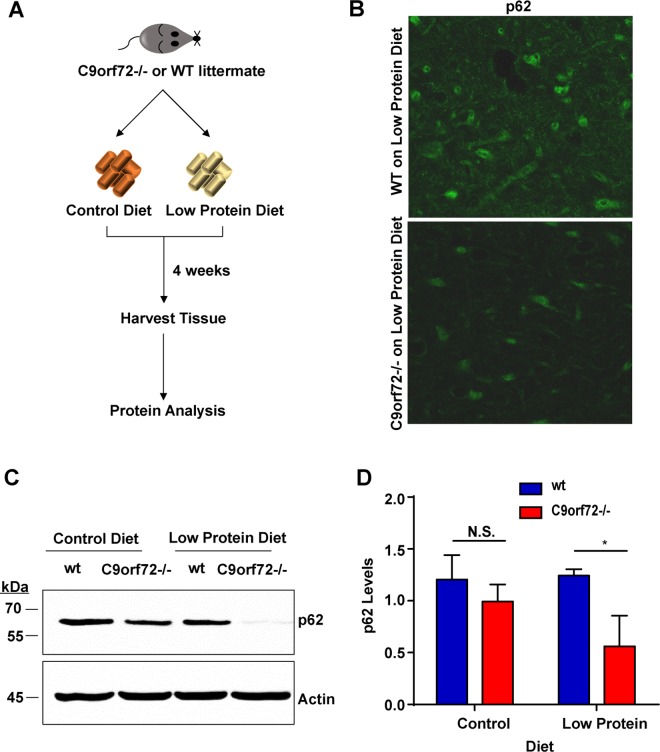
p62 is decreased in C9orf72 knockout mouse brains. **A)** Schematic representation of C9orf72 mouse tissue analysis. C9orf72-/- or wild-type mice were fed normal chow or amino acid-deficient chow (low- protein diet) for 4 weeks prior to harvesting tissue. **B)** Immunohistochemistry analysis of p62 in mouse brain. Brain of mouse on low protein diets were harvested and stained with p62 antibodies. p62 is decreased in C9orf72-/- brains compared to wild type brain, although it is expressed in the same region of brain. No p62 aggregates detected. **C)** Immunoblot analysis of brain homogenates from C9orf72 KO and wild-type animals fed control or low protein diet. C9orf72 KO mice show a decrease in p62 levels compared with wild-type littermates under the low protein diet condition. **D)** Quantification of p62 levels in brain homogenates derived from C9orf72 KO and wild-type animals fed control or low-protein diet. C9orf72 KO mice show a slight but significant decrease in p62 levels when compared with wild-type littermates under the low protein diet condition (n = 3, *p<0.05). Student’s *t* test is used and data is presented as mean ± SEM.

We next investigated whether the absence of C9orf72 alters the markers of autophagy *in vivo*. Since mTOR signaling senses nutrient stresses and autophagy induction is a natural response to nutrient stresses through mTOR, we asked whether the absence of C9orf72 affects the autophagic response under these stress conditions. We applied amino acid withdrawal by feeding mice a low-protein diet that is well-tolerated in young animals [37]. Beginning at 4 months of age, gender-matched wild-type and C9orf72 KO littermates were fed either normal or amino acid-deficient chow for four weeks before tissues were harvested for analysis (Fig 5A). We first examined autophagy in the brain of C9orf72 KO mice. Because of the low levels of LC3 conversion during starvation in the brain [38], we assessed the levels of the autophagy marker protein p62. Western blotting of brain homogenates showed a slight decrease in p62 levels in C9orf72 KO mice when compared with wild-type littermates, a defect that became more pronounced when the mice were on the low-protein diet (Fig 5C and 5D). The decrease of p62 was not due to change in its solubility since no insoluble p62 was detected in western analysis or histological examinations (Fig 5B). The lack of accumulation of p62 in the brains of C9orf72 KO mice suggests an increased autophagy activity. Consistent with the results in the mouse brain, we also observed a decrease in p62 levels in C9orf72-/- MEF lines compared with wild-type cells (S5A Fig and S5B Fig). We next examined the liver, a common tissue type used to study autophagy, harvested from the wild-type and C9orf72 KO littermates, for changes in LC3. We observed a decrease in the level of LC3II protein or relative increase of LC3I protein in C9orf72-/- livers relative to the wild-type controls under the low protein diet condition (S5C Fig and S5D Fig).

### C9orf72 interacts with DENN Protein SMCR8

**Fig 6:**
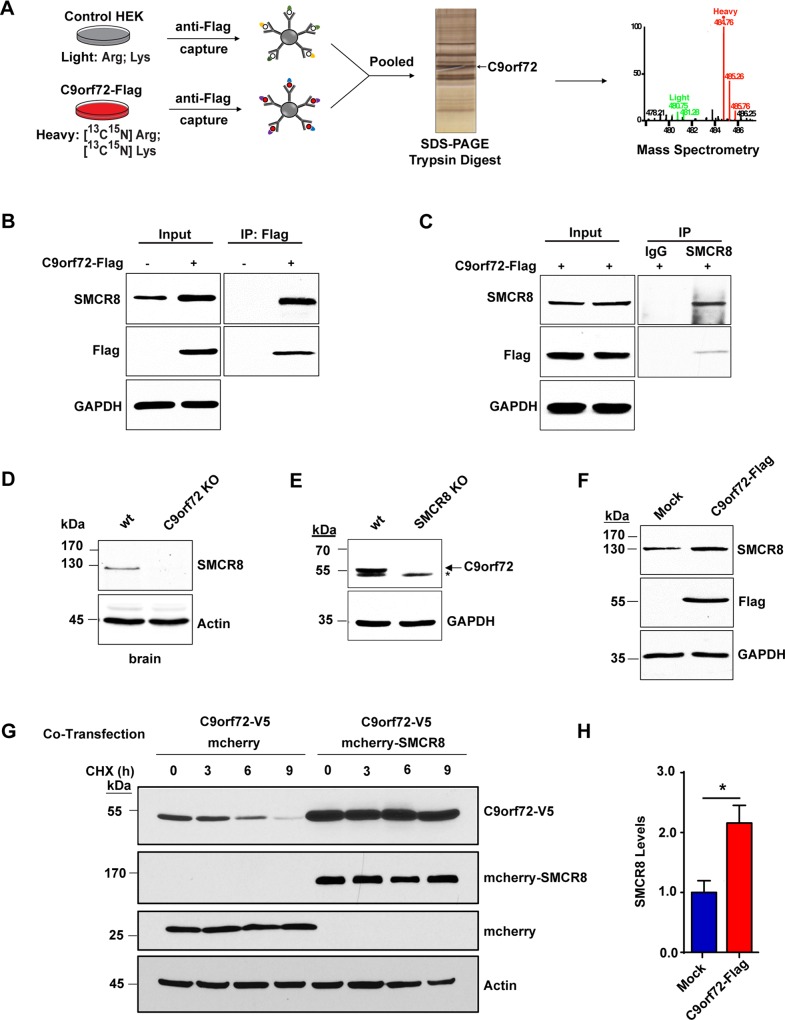
C9orf72 and SMCR8 form a stable protein complex. **A)** Schematic representation of SILAC mass spectrometry screen for C9orf72 interacting proteins. Metabolically labeled HEK293T cells expressing Flag-tagged C9orf72 Isoform A (heavy) or non-transfected control cells (light) were incubated with anti-Flag-conjugated beads and the resulting eluents were pooled, resolved on an SDS-PAGE gel, trypsin-digested and analyzed by LC-MS/MS. **B)** Immunoblot analysis of C9orf72 immunoprecipitates. HEK293T cells were either mock-transfected or transfected with C9orf72-Flag, and the protein immunoprecipitated from the cell lysates using anti-Flag-conjugated beads. The resulting eluents were probed for Flag (to detect C9orf72) and SMCR8. SMCR8 is only present in C9orf72-positive immunoprecipitates and does not bind to beads alone. **C)** Immunoblot analysis of SMCR8 immunoprecipitates. HEK293T cells were transfected with C9orf72-Flag and the resulting lysates were incubated with SMCR8 antibody and protein A Sepharose beads and the resulting immunoprecipitates analyzed via immunoblotting. SMCR8 antibody, but not control IgG, immunoprecipitated C9orf72-Flag. **D)** Analysis of SMCR8 levels in C9orf72 KO mice. Immunoblot analysis of C9orf72-/- mouse and wild-type littermate brain homogenates shows the lack of SMCR8 protein in C9orf72 KO animals compared with wild-type control animals. **E)** Analysis of C9orf72 levels in SMCR8 KO cells. Immunoblot analysis of SMCR8 KO HAP1 cell lysates shows a decrease in C9orf72 levels compared with control HAP1 cells. Arrow points to C9orf72 and asterisk indicates a cross-reacting band. **F)** Analysis of SMCR8 levels after exogenous C9orf72 expression. HEK293T cells were either mock-transfected or transfected with C9orf72-Flag and the indicated proteins analyzed via immunoblotting. **G)** Analysis of C9orf72 turnover after SMCR8 co-expression. HEK293T cells were co-transfected with C9orf72-V5 and mCherry-SMCR8, or mCherry control, expression constructs. Approximately 48 hours after transfection, cells were treated with cycloheximide, collected at the indicated time points, and analyzed via immunoblotting. Overexpression of SMCR8 dramatically stabilizes C9orf72. **H)** Quantification of the results in panel F from four independent experiments. Overexpression of C9orf72 significantly increased SMCR8 levels (n = 4, *p<0.05). Student’s *t* test is used and data is presented as mean ± SEM.

To gain molecular insight into the function of C9orf72, we performed a quantitative proteomic screen for protein interactors of the C9orf72 protein using stable isotope labeling by amino acids in cell culture (SILAC) mass spectrometry (Fig 6A). Human C9orf72 Isoform A with a C-terminal Flag tag was expressed in HEK293T cells metabolically labeled with ^13^C,^15^N L-Arginine and L-Lysine and immunoprecipitated using Flag-tag beads. A parallel immunoprecipitation was performed using unlabeled mock-transfected cells as a control to identify proteins that bound to the Flag-tagged beads alone. The resulting immunoprecipitates were pooled and analyzed via mass spectrometry to identify proteins that were enriched by the C9orf72 bait. We identified SMCR8 as the top C9orf72 interactor since it had the highest SILAC ratio or enrichment (S6A and S6B Fig and S1 Table). Notably, SMCR8, although uncharacterized, is also a DENN-like protein [20, 21]. We validated this interaction by co-immunoprecipitation, with Flag-tagged C9orf72 pulling down endogenous SMCR8 in HEK293T cells (Fig 6B). Conversely, reciprocal immunoprecipitation experiments demonstrated that an anti-SMCR8 antibody pulled down Flag-tagged C9orf72, confirming their interaction (Fig 6C). The interaction was further validated by co-immunoprecipitation of co-expressed Flag-SMCR8 and C9orf72-V5 proteins (S6C Fig). Consistently, GFP-tagged C9orf72 and mCherry-tagged SMCR8 both localized to the nucleus and the cytoplasm in HEK293T cells (S6D Fig).

### C9orf72 and SMCR8 form a stable complex

Since we identified SMCR8 as the most abundant protein interactor of C9orf72, we asked whether C9orf72 influences the level of SMCR8 protein. While examining the brain lysates from the C9orf72 KO mice, we observed a dramatic reduction in the level of SMCR8 protein. Although present in wild-type brains, SMCR8 was not detected in C9orf72-/- brain homogenates by western blotting (Fig 6D). Examination of SMCR8 transcripts by qPCR showed no reduction in its mRNA levels, supporting that C9orf72 influences SMCR8 at the protein level (S7A Fig). Notably, WD repeat-containing protein 41 (WDR41), another protein identified in our proteomic screen (S1 Table) and recently confirmed to be an interactor of the C9orf72/SMCR8 complex [28, 29], was not decreased in C9orf72-/- brain samples (S7C Fig). In addition, overexpression of C9orf72 in HEK293T cells increases SMCR8, suggesting that C9orf72 regulates SMCR8 protein levels (Fig 6F and 6H).

To further study the function of SMCR8, we obtained a CRISPR/Cas-9 generated SMCR8 KO cell line. This cell line contains a frameshift mutation in the first exon of SMCR8 resulting in the loss of the full-length protein product (S8A–S8C Fig). Since we observed that C9orf72 regulates SMCR8 protein levels, we asked whether SMCR8 reciprocally influences the levels of C9orf72. By examining the lysates from the SMCR8 KO cells by western blotting, we observed a dramatic reduction in the level of C9orf72 protein (Fig 6E). We observed the same effect on the C9orf72 protein when we treated HEK293T cells with validated SMCR8 shRNA compared with control cells transfected with a scrambled shRNA control (S8D and S8E Fig). Examination of C9orf72 transcripts in SMCR8 KO cells by qPCR showed an increase in its mRNA levels (S7B Fig), suggesting that the loss of SMCR8 decreased the C9orf72 protein level not by reducing its RNAs.

Next we studied how SMCR8 regulates C9ORF72 protein levels. We first asked whether the regulation occurs due to changes in protein stability or turnover. Since the level of C9ORF72 was too low to allow for chase experiments to probe their turnover in the SMCR8 KO cells, we overexpressed C-terminal-V5 tagged C9orf72 and N-terminal-mCherry tagged SMCR8, or an mCherry only control, into HEK293T cells and studied their protein levels. Compared with the mCherry control, the expression of mCherry-SMCR8 substantially increased the level of C9orf72-V5 (Fig 6G). Importantly, under a 12-hr chase condition after treatment of the cells with the translation inhibitor cycloheximide, mCherry-SMCR8 dramatically stabilized the co-expressed C9orf72-V5 as compared with the mCherry control (Fig 6G). We also confirmed that the C9orf72-V5 protein was degraded through both proteosomal and lysosomal pathways, since inhibition of proteasomal degradation by MG132 treatment or inhibition of lysosomal degradation by Bafilomycin treatment stabilized the C9orf72-V5 protein (S7E Fig). These data indicate that C9orf72 and SMCR8 form a stable cognate protein complex that protects C9orf72 from degradation.

### SMCR8 regulates mTOR signaling and autophagy

**Fig 7:**
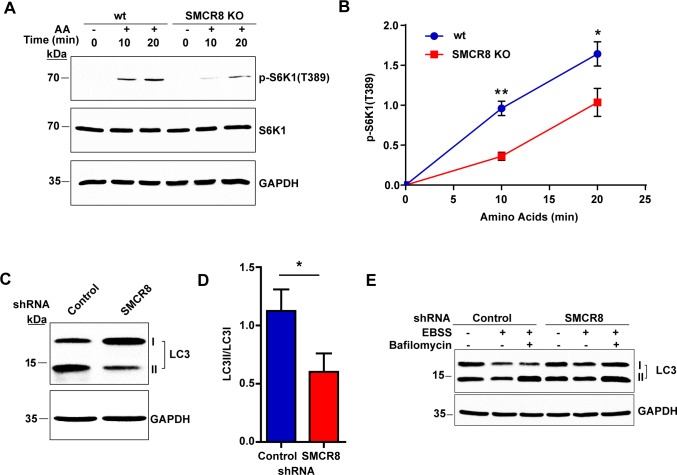
SMCR8 regulates mTOR signaling and autophagy. **A)** Immunoblot analysis of mTOR activity after starvation and amino acid stimulation. HAP1 control and SMCR8 KO cells were starved for 50 minutes and supplemented with amino acids for 10–20 min before lysate collection. The mTOR activity was assessed via immunoblotting for the phosphorylation of its downstream substrate S6K1. **B)** Quantification of p-SK61 (T389) levels after starvation and amino acid stimulation in control and SMCR8 knockout cells from three independent experiments. Knockout of SMCR8 significantly decreased p-S6K1 levels compared with wild-type cells (n = 3, *p<0.05, **p<0.005). **C)** Immunoblot analysis of LC3 levels after shRNA-mediated knockdown of SMCR8 in HEK293T cells. HEK293T cells were transfected with SMCR8 or control shRNA and lysates collected 72 hours after transfection. The indicated proteins were detected by immunoblotting of the lysates using an antibody against LC3. **D)** Quantification of the LC3II to LC3I ratio obtained after shRNA-mediated knockdown of SMCR8 in HEK293T cells from three independent experiments. Knockdown of SMCR8 significantly decreased the ratio of LC3II to LC3I compared with control cells (n = 3, *p<0.05). **E)** Immunoblot analysis of LC3 levels before and after autophagy induction with nutrient starvation. HEK293T cells were transfected with SMCR8 shRNA or scrambled shRNA control. Approximately 72 hours after transfection, cells were treated with starvation medium (EBSS) with and without Bafilomycin for 2 hours and the resulting lysates were analyzed via immunoblotting for LC3. Student’s *t* test is used and data is presented as mean ± SEM.

Given the connections between C9orf72 and SMCR8, we asked whether loss of SMCR8 plays a role in mTOR signaling similar to that of C9orf72. In accordance with the results from C9orf72-/- MEF cells, knockout of SMCR8 led to a similar defect. In SMCR8 KO HAP1 cells, the phosphorylation of S6K1 after amino acid treatment was significantly decreased when compared with control cells (Fig 7A and 7B).

Next, we investigated if loss of SMCR8 also affected autophagy. First, we examined LC3 levels after shRNA-mediated knockdown of SMCR8 in HEK293T cells by immunoblotting. As observed in C9orf72-/- MEFs and C9orf72 shRNA treated HEK293T cells, knockdown of SMCR8 led to a decrease in the ratio of LC3II to LC3I, when compared with cells treated with scrambled shRNA (Fig 7C and 7D). Additionally, Bafilomycin treatment of the cells under starvation showed a similar accumulation of LC3II with the SMCR8 knockdown as that of the control cells (Fig 7E). Thus, the autophagic flux appears to be intact in the absence of SMCR8 in this cell line.

## Discussion

In the present study, we have identified a function of C9orf72 in regulating mTOR signaling and autophagy. Loss of C9orf72 leads to deficiency in the phosphorylation of S6K1 and increase of TFEB protein levels and nuclear activity, demonstrating a regulatory role of C9orf72 in the mTOR signaling pathway upstream of autophagy.

We identified the major interacting partner of C9orf72 protein as SMCR8. The most structurally homologous proteins to SMCR8 and C9orf72 in the human proteome are folliculin (FLCN) and folliculin-interacting proteins (FNIP1 or 2), respectively [20, 21]. Like SMCR8 and C9orf72, FNIP and FLCN are DENN domain-containing proteins [20, 21] that interact with each other in a protein complex [29], that have also been shown to regulate autophagy and mTOR signaling [30, 31]. Since the FNIP and FLCN complex was shown to function as either GAP or GEF for the Rag GTPases in the mTORC1 pathway, we speculate that the C9orf72-SMCR8 complex may function in a similar fashion in autophagy and mTOR signaling.

Our results demonstrate that loss of C9orf72 can alter the dynamics of autophagy. We observed a relative increase in LC3I levels upon loss of C9orf72 (S2 Fig), in consistence with a recent report for LC3 levels in C9orf72 KO mouse liver and spleen tissues [39], which we interpret as an increase in autophagosome turnover instead of a decrease in LC3II formation. In support of this model, we did not observe a decrease in LC3II levels after Bafilomycin treatment under full nutrient conditions, suggesting that the formation of LC3II is intact (S3 Fig). Moreover, we observed increased autophagic flux in response to nutrient deprivation in C9orf72-/- cells (Fig 4). Consistent with our model of increased autophagic flux, we observed a loss of mTOR activity after loss of C9orf72, which is classically associated with increases in the autophagic pathway. In support of our finding, a recent study showed decreased mTOR signaling in C9orf72-depleted HeLa cells [40]. Importantly, we observed a substantial increase of TFEB and its lysosomal targets in C9orf72 knockout mice (Fig 3). As a master regulator of lysosome biogenesis, TFEB is known to promote cellular lysosomal capacity and autophagy [32]. Consistent with our findings, we also observed a decrease in levels of the autophagy receptor p62 in brain tissues from C9orf72 KO mice and observed a similar decrease in the C9orf72 KO MEFs. Interestingly, it was recently reported that loss of the SMCR8 homologue folliculin similarly results in decreased mTOR signaling and a TFEB-mediated enhancement of the lysosomal compartment [31].

There have been recent reports describing C9orf72’s functions in autophagy [39, 41–43], including a decrease in autophagy initiation as a result of knockdown of C9orf72 [41, 42]. These observations are not necessarily mutually exclusive to our present study. C9orf72 might play a multifunctional role in different steps of the autophagic pathways. While C9orf72 may influence the function of the FIP200/ULK1 autophagy initiation complex [41, 42], it could also regulate mTOR signaling and TFEB and thus promote autophagic flux, as observed in the present study. Furthermore, the manifestation of the phenotypes could be influenced by the dynamic nature and condition-dependent activity levels of autophagy pathways. Due to the reduced state of mTOR signaling in C9orf72-depleted cells, the increased autophagic flux of these cells could be more readily revealed under nutrient deprivation conditions, as employed in the present study. Notably, the autophagy receptor p62 is both a substrate of autophagy and a transcriptional target of TFEB [44], therefore it is subject to opposing regulation by upregulation of TFEB. Taken together, our study provides evidence that long-term loss of C9orf72 leads to physiological changes that are characterized by reduced mTOR activity, in consistence with increased TFEB signaling leading to enhanced cellular lysosomal capacity and autophagic flux.

Since multiple studies have reported that the hexanucleotide repeat expansion led to reduced expression of C9orf72 mRNAs and proteins in patient cells and brains [11–15], the defects associated with loss of C9orf72 protein function could contribute to the pathogenesis of relevant neurodegenerative diseases. Several studies have reported that neither mice lacking C9orf72 protein nor those expressing the human C9orf72 gene containing the HRE mutation exhibited major neuronal loss [17, 45–47], with the exception of one study reporting neurodegeneration in transgenic mice expressing HRE-containing C9orf72 [48]. Our observation that C9orf72 ablation changes LC3 levels in motor neuron cultures suggests that loss of C9orf72 might affect neuronal functions. Autophagy and nutrient sensing are essential for neuronal health and their alteration is an increasingly recognized feature in aging-related neurodegenerative diseases [49, 50]. Of note, several autophagy-related genes, including p62, optineurin, and TBK1, have been linked to ALS [51–53]. Proteinaceous inclusions positive for p62 are a pathologic feature in brains from patients carrying the C9orf72 HRE mutation [54]. Taken together, our findings suggest that C9orf72 protein has a function in the metabolic processes of the cell and reduction in its function may contribute to related age-dependent neurodegenerative diseases.

## Materials and Methods

### Ethics Statement

The animal protocol (MO15M165) was approved by the Johns Hopkins Animal Care and Use Committee following the National Research Council’s guide to the Care and Use of Laboratory Animals.

### DNA and shRNA plasmids

C9orf72 cDNA (HsCD00398737) was obtained from Arizona State University and SMCR8 cDNA (HsCD00347993) from Harvard Plasmid Repositories. The C9orf72 constructs were generated using the Gateway cloning system (ThermoFisher, Waltham, MA) with a C-terminal 3xFlag or V5 tag. The SMCR8 constructs were generated with an N-terminal Flag or mCherry tag using Gateway or classical cloning methods, respectively. All shRNAs were cloned into the pRFP-C-RS vector (Origene), which was modified to remove the RFP coding sequence via digestion with *Mlu*I and *Bgl*II followed by blunting and religation. The following shRNA sequences were used: 5’ctgtgttacctcctgaccagtcagattga 3’ (SMCR8); 5’cttccacagacagaacttagtttctacct 3’ (C9orf72). The autophagy luciferase assay plasmids were kindly provided by Brian Seed (Harvard) and the normalization plasmid pCMV-SEAP was from Addgene (24595, Alan Cochrane, University of Toronto). GFP-TFEB was obtained from Addgene (38119, Shawn Ferguson, Yale University). GFP-TFEB used for MEF experiments was described before [55]. For GFP-LC3, human LC3 was cloned into pEGFP-C1. RFP-Rab7 was generated from EGFP-Rab7 (a kind gift from Bo van Deurs at University of Copenhagen) by exchanging EGFP into RFP.

### Animals

Mouse ES cell lines containing a heterozygous allele of 3110043O21Rik^tm1.1(KOMP)Mbp^ were obtained from the KOMP repository. The ES cells with a strain background of C57BL/6N-Atm1Brd were microinjected into blastocysts, and the germline-transmitted allele was maintained on the C57BL/6 background. Male mice bearing the original targeting allele were crossed with SOX2-Cre recombinase transgenic female mice (Jackson Laboratory, 008454) to remove the LoxP-flanked neomycin selection cassette. The resulting allele was bred to heterozygotes and homozygotes that were used in this study. The genotyping primers were the following: gaatggagatcggagcacttatgg (wild-type, forward), gccttagtaactaagcttgctgccc (wild-type, reverse), gcacaagctatgttcatttgg (KO, forward), gactaacagaagaacccgttgtg (KO, reverse).

### Mouse Tissue and Survival Analysis

For the low-protein diet assay, 16 week old, gender-matched littermates were fed a low-protein diet (Test Diet 5767, 5% protein) or standard chow for 4 weeks prior to tissue collection. Mouse tissue was lysed in modified RIPA buffer (50 mM Tris pH 6.8, 150 mM NaCl, 0.5% SDS, 0.5% Sarkosyl, 0.5% NP40, 20 mM EDTA, Roche protease inhibitors) using a Dounce homogenizer, sonicated, and used for further analysis. For the survival analysis, Kaplan-Meyer curves were generated using GraphPad Prism software.

### Cell Culture and DNA Transfection

All cells were maintained in DMEM supplemented with 10% FBS unless otherwise noted. The SMCR8 knockout HAP1 cells (HZGHC003606c011) were created at Horizon Genomics (Vienna, Austria) by using CRISPR/Cas9 and maintained in IMDM supplemented with 10% FBS. All cell lines were cultured in 95% O2/5% CO_2_. Cell lines were transfected using Lipofectamine 2000 (ThermoFisher) according to the manufacturer’s instructions.

### Mouse Embryonic Fibroblasts and Stem Cells

Mouse embryonic fibroblasts were isolated from Day 13 embryos by trypsin digestion and their genotypes confirmed by PCR. The lines were immortalized by transfecting cells with the SV40-T antigen-expressing plasmid pSG5 Large T using Lipofectamine 2000. The cells were passaged at least 5x to ensure the homogeneity of the cell population before use in experiments. To isolate embryonic stem cells, 14-week old C9orf72 heterozygous females were treated with Pregnant Mare Serum Gonadotropin via intraperitoneal injection followed by injection 24 hours later with human chorionic gonadotrophin to induce superovulation prior to mating with C9orf72 heterozygous males. Embryos were collected 48 hours after the second injection at the transgenic core facility at Johns Hopkins University and the genotypes confirmed by PCR. Wild type and C9ORF72-/- ES cells were cultured on 0.1% gelatin coated plates in 2i media consisting of half of DMEM/F12 and half of Neurobasal media containing N2-supplement (ThermoFisher Scientific 17502048), B-27 supplement (ThermoFisher Scientific 17504044), 0.05% BSA (ThermoFisher Scientific 15260037), 50 units Penicillin-Streptomycin, 1 μM PD03259010 (Stemgent 04–0006), 3 μM CHIR99021 (Stemgent 04–0004), 2 mM Glutamine, 150 μM Monothioglycerol (Sigma M6145) and 1,000 U/ml LIF.

### Motor Neuron Cultures

Motorneuron differentiation protocol was modified from a previously reported induction protocol using retinoic acid and Smoothened agonist (SAG, Millipore) [56]. Briefly, 1 X 10^6^ ES cells were harvested by dissociating with 0.05% trypsin-EDTA (ThermoFisher) and cultured in suspension condition in DFK5 media (DMEM/F12 based media containing 5% knockout serum replacement, 1 x insulin transferrin selenium (ThermoFisher), 50 μM nonessential amino acids, 100 μM β-mercaptoehanol, 5 μM thymidine, 15 μM adenosine, 15 μM cytosine, 15 μM guanosine and 15 μM uridine) for 48 hours. After two days, the resulting embryonic bodies were treated with 2 μM retinoic acid and 600 nM of SAG in fresh DFK5 media and cultured another 4 days. Media was replaced every two days. For experiments, 1.5 x 10^6^ cells were plated on each well of laminin-coated 6 well plates in DFK5 media containing 5 ng/mL glial-derived neurotrophic factor (GDNF; Peprotech), 5 ng/mL brain-derived neurotrophic factor (BDNF; Peprotech), 5 ng/mL neurotrophin-3 (NT-3; Peprotech) for 24 hours. After 24 hours, media were changed with DFKNB media consisting of half of DFK5 media and half of Neurobasal media with B27, 5 ng/mL GDNF, 5 ng/mL of BDNF and 5 ng/mL of NT-3.

### mTOR Nutrient Sensing Assay

All cells were starved using Earles’s balanced salt solution (EBSS; Sigma) for 50 min. Amino acid stimulation was applied by treating cells with essential amino acids (Gibco) and non-essential amino acids (Quality Biologicals) in EBSS. Amino acids were diluted to match DMEM concentrations. Cells were treated in EBSS plus amino acids for 10–20 min prior to lysate collection. Lysates were processed as described above except that Phospho-stop inhibitor tablets (Roche) were added to the lysis buffer.

### LC3 and Rab7 colocalization assay

Wild type or C9orf72-/- MEF cells were transfected with GFP-LC3 and RFP-Rab7, and cells were treated with DMEM containing 10% FBS (full medium; FM) or EBSS (nutrient deprivation; ND), in the presence or absence of the lysosomal inhibitor Bafilomycin A1 (Baf) for 3 hours, before being fixed with 4% paraformaldehyde. High resolution images were acquired using a Z sweep function, permitting acquisition of total cellular fluorescence using a DeltaVision Elite microscope (GE Healthcare) with 60× PlanApo NA 1.4 Oil objective lens (Olympus) and images were deconvolvd using SoftWoRx software. Subsequently, individual cells were manually segmented; LC3-positve vesicles in the green channel and Rab7-positive vesicles in the red channel. Using a boolean function, the overlap between these segmented images was used to generate a third mask corresponding to co-localized LC3 and Rab7 vesicles (Yellow image) which are autolysosomes. Single vesicle areas were calculated from LC3, Rab7 and co-localization masks, and mean values for each experiment were normalized to ND or FM, as indicated.

### Florescence microscopy

HEK293T cells were grown on class coverslips and transfected with the indicated constructs as described above. Images were captured using an SP8 confocal microscope (Leica) and processed using ImageJ software. For GFP-TFEB imaging in HEK293T cells, live cells were imaged while maintained in phenol red free DMEM containing 10% FBS. For LysoTrackerBlue staining, 50nM of LysoTrackerBlue was added into the media for an hour and the media was changed before imaging.

### SDS-PAGE and Immunoblotting

Cells were collected in modified RIPA lysis buffer (50 mM Tris pH 6.8, 150 mM NaCl, 0.5% SDS, 0.5% Sarkosyl, 0.5% NP40, 20 mM EDTA, Roche protease inhibitors) and sonicated using a Diagenode Bioruptor for 15 min (high setting, 30 sec pulse, 3x 5 min) and the resulting lysates were centrifuged at 16,000 x g for 10 min at 4°C. For mTOR assays, cells were collected in HEPES lysis buffer (40 mM HEPES pH 7.4, 2 mM EDTA, 1% Triton, Roche protease and PhosphoStop inhibitors) and centrifuged at 16,000 x g for 10 min at 4°C. Protein concentrations were determined using the bicinchonic acid assay (ThermoFisher). For GFP-TFEB nuclear import analysis, cells were fractionated using Subcellular Fractionation Kit for Cultured Cells (ThermoFisher) following the manufacturer’s protocols. Then the cytoplasmic and membrane proteins were combined as the cytosolic fraction and the nuclear soluble and chromatin bound proteins were combined as the nuclear fraction. PARP was used as a nuclear marker and Caspase 3 as a cytoplasmic marker. For autophagic flux determination, wild type and C9orf72-/- MEFs were subjected to fresh fully supplemented medium or nutrient deprivation (EBSS) for 3 hours. Antibodies used were: mouse anti-Flag, rabbit anti-SMCR8 (Sigma), mouse anti-GFP, rabbit anti-GAPDH (ThermoFisher), rabbit anti-C9orf72, mouse anti-actin (Santa Cruz), rabbit anti-p62, rabbit anti p-70S6K, rabbit anti p-p70S6K, rabbit anti-PARP, rabbit anti-Caspase 3, rabbit anti-DYKDDDDK (Cell signaling), rabbit anti-V5 (Novus), mouse anti-V5 (Invitrogen), mouse anti-TFEB (Mybiosources), mouse anti-Lamp1 (Hybridoma Bank; #H4A3-s), mouse anti-Lamp2 (Hybridoma Bank, #H4B4-s), and rabbit anti-LC3B (Abcam).

### Quantitative Mass Spectrometry by SILAC

HEK293T cells were incubated in heavy (^13^C_6_,^15^N_4_ L-Arginine, ^13^C_6_,^15^N_2_ L-Lysine) or light (^12^C_6_,^14^N_4_ L-Arginine, ^12^C_6_,^14^N_2_ L-Lysine) DMEM and verified for near-completion of labeling by mass spectrometry. Heavy isotope-labeled cells were transfected with C9orf72-Flag and light isotope-labeled cells were mock transfected with Lipofectamine. After immunoprecipitation with Flag-tag beads, the resulting immunoprecipitates were pooled, concentrated, separated via SDS-PAGE, and subjected to trypsin in-gel digestion. The digested samples were subjected to LC-MS/MS analysis on an Orbitrap Elite mass spectrometer coupled with Easy nLC II liquid chromatography system. The mass spectrometry data were analyzed using the Proteome Discoverer 1.4 software suite against human Refseq 59 protein database. A 1% peptide-spectrum-match and peptide-level false discovery rate was applied for data analysis.

### Immunoprecipitations

Cells were lysed in IP buffer (50 mM Tris pH 7.4, 150 mM NaCl, 1% Triton, Roche protease inhibitors), incubated for 30 min on ice and centrifuged at 16,000 x g prior to immunoprecipitation. For Flag immunoprecipitations, the resulting supernatants were added to Flag-conjugated beads (Sigma, St. Louis, MO) and incubated for 2 h at 4°C with gentle rotation. The beads were washed 5x with IP buffer and the immunoprecipitates eluted by incubating the beads with SDS-PAGE loading dye for 5 min at 95° C. For SILAC analysis, immunoprecipitates were eluted using Flag peptide (Sigma) at 5μg/μl. For SMCR8 immunoprecipitation, anti-SMCR8 antibody (Abcam) was incubated with protein A Sepharose beads (BioRad, Hercules, CA) and incubated at room temperature for 2 h and the beads treated as described above.

### ATG4B Luciferase Assay

The ATG4B-dependent processing of LC3 in autophagy was quantified with a *Gaussia* luciferase release assay [57, 58]. ATG4B-induced proteolytic cleavage of an actin-anchored LC3-luciferase fusion protein (Act-LC3-Gluc) releases the Gluc fragment and enables its secretion into the cell medium. Cells were transfected with shRNA or scrambled control and Act-LC3-Gluc or control Act-Gluc plasmid together with the secreted alkaline phosphatase normalization control, CMV-SEAP. Cell medium (150 μl) was withdrawn 48–72 h after transfection and the luciferase and SEAP in the medium were analyzed by using *Gaussia* luciferase assay kit (New Englabnd Biolabs) and the Phospha-light SEAP reporter system (ThermoFisher) using a microplate reader (Synergy H1, Bio-Tek).

### Immunohistochemistry

Gender matched four month old mice were intracardially perfused with ice-cold 4% paraformaldehyde. Brains were removed and post-fixed and equilibrated with 30% sucrose. Sections were prepared using Cryostar NX70 (ThermoScientific). Sections were washed with PBST three times to permeabilize cells, pre-incubated with 10% anti-goat serum for an hour at RT, incubated with an anti-p62 antibody (Cell Signaling) for overnight at 4°C, and then incubated with Alexa488-congugated secondary antibody after washing three times with PBS. Images were obtained using an SP8 confocal microscope (Leica) after samples were washed three times with PBS and mounted with Vectashield.

### Quantitative RT-qPCR

Total RNA was isolated from cells with the RNeasy Plus Mini kit and cDNAs were synthesized with the QuantiTect reverse transcription kit (Qiagen). Primers for quantitative RT-qPCR were from PrimerBank unless otherwise noted (S2 Table). RT-qPCRs were performed on a BioRad thermal cycler with iQ SYBER Green PCR mix (BioRad).

### Quantitation and Statistical Analysis

All quantitation and statistical tests were performed using ImageJ and GraphPad Prism software (Version 6.0). The p-values for all experiments were obtained using Student’s *t* tests unless indicated otherwise.

## Supporting Information

S1 Fig Human vs mouse C9orf72 protein alignment. Alignment depicting the predicted two isoforms of human C9orf72 and the three isoforms of mouse C9orf72. The accession numbers are as follows: NP_00124293 Isoform A Human; NP_659442 Isoform B Human; EDL05456 Isoform 1 Mouse; Q6DFW0.1 Isoform 2 Mouse; EDL05457 Isoform 3 Mouse.
(TIF)

S2 Fig LC3 protein levels in C9orf72 knockout cells. **A)** Immunoblot analysis of whole protein lysates from representative MEF lines generated from C9orf72 KO and wild-type littermates using an antibody against LC3. C9orf72-/- MEFs show an increase in the ratio of LC3I to LC3II when compared with wild type cells. **B)** Quantification of the LC3II to LC3I ratio from representative C9orf72-/- and wild-type MEF lines from three independent experiments. C9orf72-/- MEFs show a significant increase in the ratio of LC3II to LC3I when compared with wild-type littermate controls (n = 3, *p<0.05). **C)** Immunoblot analysis of neurally differentiated cells derived from C9orf72 KO and wild-type embryonic stem cells. C9orf72-/- cells with enriched motor neurons show a dramatic increase in LC3I compared with wild-type controls. Student’s *t* test is used and data is presented as mean ± SEM.
(TIF)

S3 Fig Analysis of autophagy flux upon loss of C9orf72. **A)** Representative live cell images of RFP-Rab7/GFP-LC3 co-localization in C9orf72-/- MEFs. RFP-Rab7 and GFP-LC3 were transfected in wild-type or C9orf72-/- cells and treated with Bafilomycin in fully supplemented medium (FM) conditions. **B)** Representative image of western blot analysis of LC3 in fully supplemented medium conditions. **C)** Immunoblot analysis of LC3 levels before and after autophagy induction with nutrient starvation. HEK293T cells were transfected with C9orf72 shRNA or scrambled shRNA control. Approximately 72 hours after transfection, cells were treated with starvation medium (EBSS) with or without Bafilomycin for 2 hours and the resulting lysates were analyzed via immunoblotting. Scale bars: 10 μm.
(TIF)

S4 Fig Luciferase release assay for ATG4B activity. **A)** Diagram depicting the luciferase release assay. This assay monitors the cleavage of LC3 by the autophagy-associated protease ATG4B. An actin-LC3-GLuc fusion protein, which is fused to the cytoskeleton, is expressed along with a constitutively secreted alkaline phosphatase (SEAP) normalization control. The cleavage of LC3 by ATG4B allows for the rapid secretion of the GLuc luciferase into the cell medium where it can be measured using standard luciferase assay methods. **B)** Quantification of ATG4B activity after shRNA-mediated knockdown of C9orf72. HEK293T cells were co-transfected with scrambled or C9orf72 shRNA, an ATG4B activity luciferase reporter, and a normalization control construct. Graph represents the cumulative luciferase signal from time points taken at 48 and 72 hours post-transfection from three independent experiments. Knockdown of C9orf72 significantly decreased secreted luciferase signal which corresponds to a decrease in ATG4B activity (n = 3, ***p<0.0005). **C)** Analysis of ATG4B protein levels after shRNA-mediated knockdown of C9orf72 in HEK293T cells. HEK293T cells were transfected with C9orf72 or control shRNA and lysates collected 72 hours after transfection and the indicated proteins detected by immunoblotting. **D)** Quantification of ATG4B protein levels after shRNA-mediated knockdown of C9orf72 in HEK293T from three independent experiments. Knockdown of C9orf72 does not significantly change ATG4B levels (n = 3). Student’s *t* test is used and data is presented as mean ± SEM.
(TIF)

S5 Fig Altered autophagy markers in C9orf72 knockout mice and MEFs. **A)** Representative immunoblot of p62 protein levels in C9orf72-/- MEFs and wild-type control cells. **B)** Quantification of p62 protein levels shows a decrease in the C9orf72-/- MEFs (n = 3, *p<0.05). **C)** Immunoblot analysis of liver homogenates from C9orf72-/- and wild-type animals fed a low-protein diet. C9orf72 KO mice show a decrease in LC3II compared with wild-type littermates. **D)** Quantification of LC3II levels in liver homogenates derived from C9orf72 KO and wild-type animals fed a low-protein diet. C9orf72 KO mice shows a significant decrease in LC3II when compared with wild-type littermates (n = 2, *p<0.05). Student’s *t* test is used and data is presented as mean ± SEM.
(TIF)

S6 Fig Analysis of the SMCR8 and C9orf72 Interaction. **A)** Representative MS spectrum of an identified peptide, ANELASVEK for SMRC8. The full MS spectrum of light and heavy forms of the peptide m/z 480.75 and m/z 484.76 and their relative intensity was shown in the spectrum. **B)** The MS/MS spectrum of the identified spectrum shown in panel (A). **C)** Validation of the C9orf72-SMCR8 interaction via co-expression in HEK293T cells. HEK293T cells were either co-transfected with Flag-tagged SMCR8 and V5-tagged C9orf72 or transfected with C9orf72-V5 alone and the resulting lysates were incubated with anti-Flag-conjugated beads. V5-tagged C9orf72 successfully co-immunoprecipitates with Flag-SMCR8 and does not bind to beads alone. **D)** Colocalization of C9orf72 and SMCR8 in HEK293T cells. Cells were transfected with GFP-tagged C9orf72 and mCherry-tagged SMCR8 and imaged using confocal microscopy. Arrow points to a cell expressing both constructs.
(TIF)

S7 Fig Analysis of the C9orf72-SMCR8 protein complex. **A)** qPCR analysis of SMCR8 RNA levels in mouse brain. RNA was isolated from wild-type or C9orf72 KO littermates and SMCR8 transcript levels were assessed by qPCR. No significant change in transcript levels were observed between samples (n = 2) **B)** qPCR analysis of C9orf72 RNA levels in HAP1 SMCR8 knockout cells. RNA was isolated from control or SMCR8 KO cells and C9orf72 transcript levels assessed by qPCR. No decrease in transcript levels was observed in SMCR8 KO cells as compared to control (n = 2, *p<0.05). **C)** Immunoblot analysis of C9orf72-/- mouse and wild-type littermate brain homogenates shows no change in WDR41 protein levels in C9orf72 KO animals compared with wild-type control animals. **D)** Immunoblot analysis of control and SMCR8 KO HAP1 cells shows no change in WDR41 protein levels. **E)** Analysis of C9orf72 protein levels after MG132 or Bafilomycin treatment. HEK293 cells were transfected with C9orf72-V5 and treated with MG132 (5 μM) or Bafilomycin (100 nM) for 16 hours and the indicated proteins analyzed by immunoblotting. C9orf72 protein levels were increased after MG132 or Bafilomycin treatment. Student’s *t* test is used and data is presented as mean ± SEM.
(TIF)

S8 Fig Validation of SMCR8 knockout and knockdown cells. **A)** Schematic representation of the CRISPR/Cas9 generated SMCR8 KO cell line. HAP1 cells were engineered to contain a 7-base pair deletion in exon 1 of the SMCR8 gene. This mutation is predicted to have an early stop codon and result in the loss of the full-length protein product. **B)** Validation of SMCR8 deletion. Sequencing results of genomic DNA extracted from control and SMCR8 knockout HAP1 cells confirming the presence of the 7-base pair deletion in exon 1. **C)** Immunoblot analysis of HAP1 cells. Immunoblot analysis of control and SMCR8 KO HAP1 cells using an antibody against SMCR8. We interpret the band detected in the knockout cells to be a cross-reactive protein product. **D)** Analysis of C9orf72 levels in SMCR8 knockdown cells. HEK293T cells were transfected with control shRNA or SMCR8-targeted shRNA and the resulting lysates analyzed via immunoblotting. Cells treated with SMCR8 shRNA show a decrease in C9orf72 levels when compared to cells treated with scrambled shRNA. **E)** Validation of SMCR8 shRNA. HEK293T cells were co-transfected with mCherry-tagged SMCR8 and either control shRNA or SMCR8 shRNA and the resulting lysates were analyzed by immunoblotting. SMCR8-targeted shRNA successfully knocked down overexpressed SMCR8, validating our shRNA.
(TIF)

S1 Table List of hits from the mass-spectrometry analysis of C9orf72-flag immunoprecipitates using stable isotope labeling with amino acid in cell cultures (SILAC). The top hits with the heavy/light SILAC ratio more than five are shown.
(PDF)

S2 Table List of quantitative PCR primers. The DNA primer sequences used for quantitative PCR are provided.
(PDF)

S1 Movie Representative behavior of an end stage C9orf72-/- mouse. A video of a representative C9orf72-/- mouse at end stage showing lethargic and weak behavior.
(MOV)
