# Supplementary figures and images for: Loss of C9orf72 Enhances Autophagic Activity via Deregulated mTOR and TFEB Signaling
Source: PLoS Genet. 2016 Nov 22;12(11):e1006443. doi: 10.1371/journal.pgen.1006443 (PMC5119725; doi:10.1371/journal.pgen.1006443)

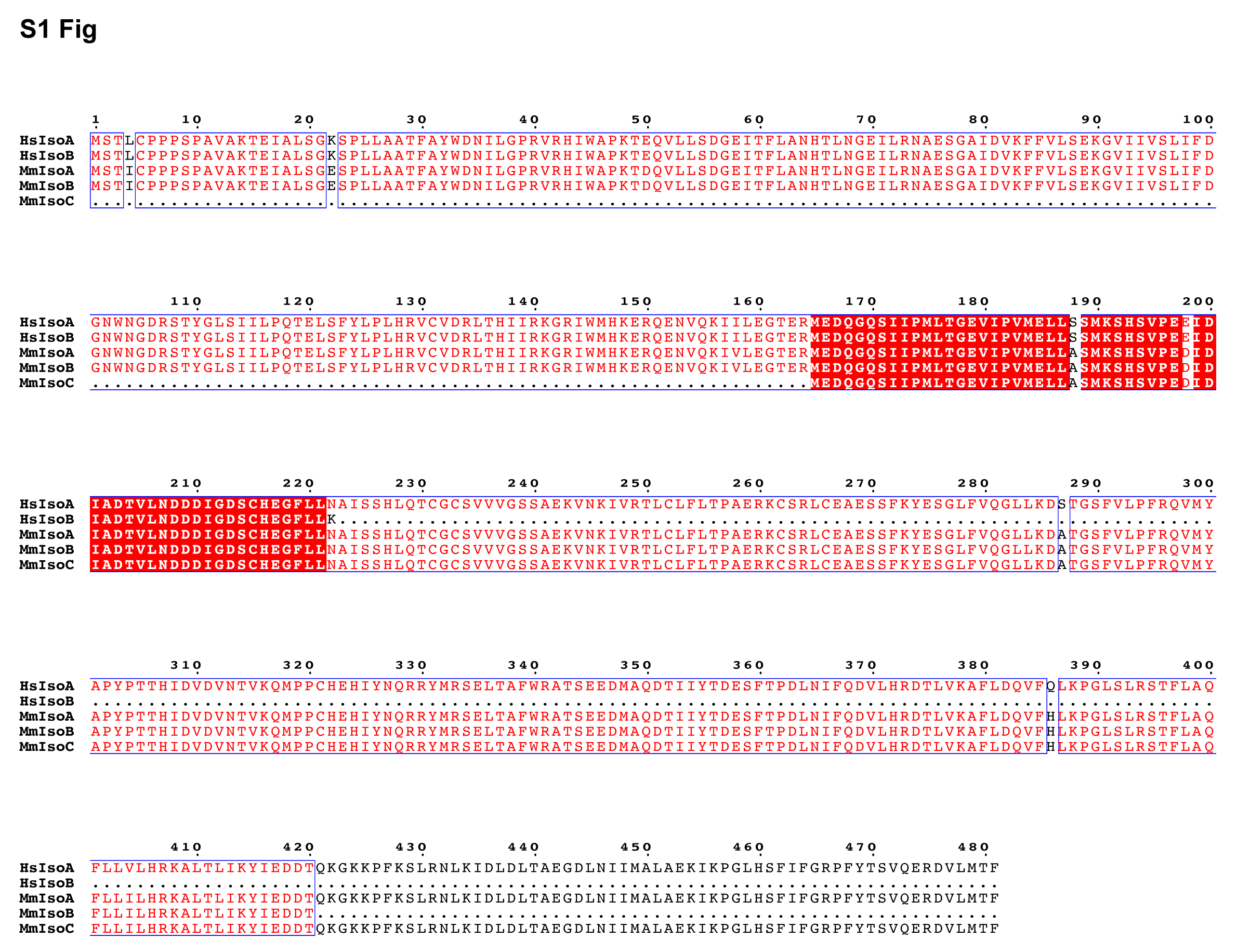

Supplement: S1 Fig — Alignment depicting the predicted two isoforms of human C9orf72 and the three isoforms of mouse C9orf72. The accession numbers are as follows: NP_00124293 Isoform A Human; NP_659442 Isoform B Human; EDL05456 Isoform 1 Mouse; Q6DFW0.1 Isoform 2 Mouse; EDL05457 Isoform 3 Mouse. (TIF) [file pgen.1006443.s001.tif]

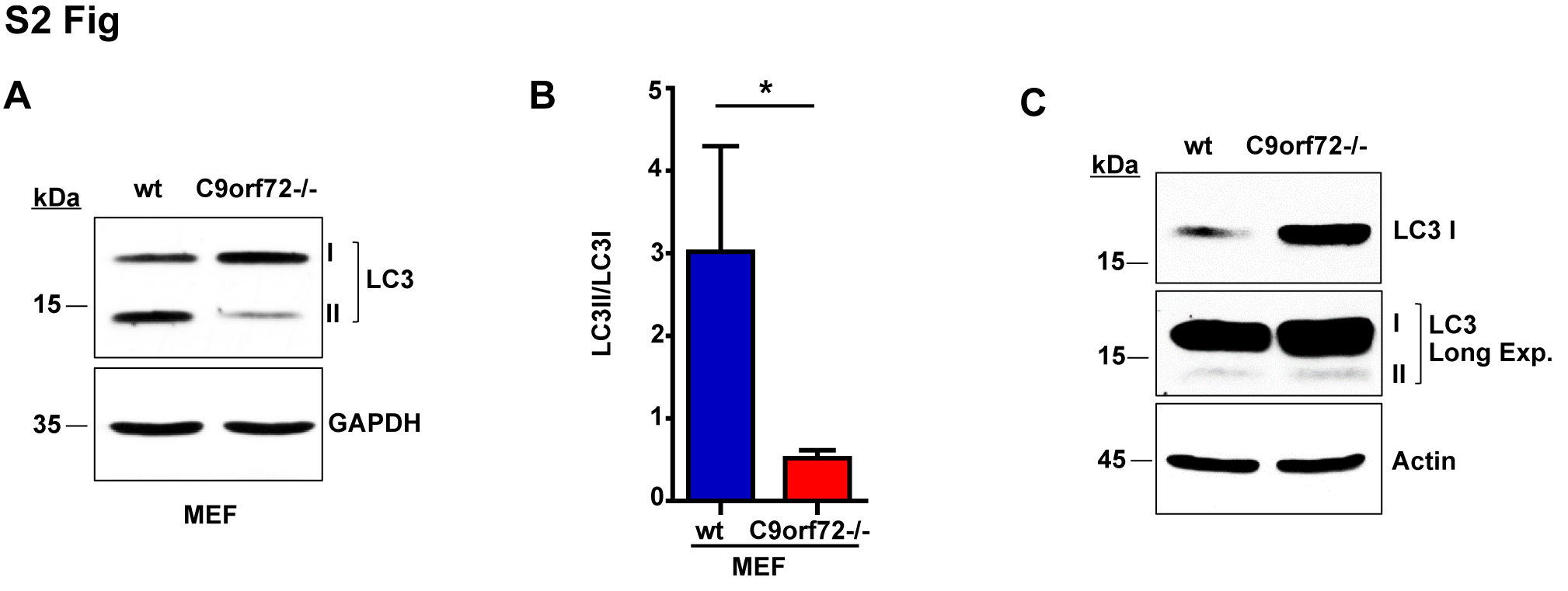

Supplement: S2 Fig — A) Immunoblot analysis of whole protein lysates from representative MEF lines generated from C9orf72 KO and wild-type littermates using an antibody against LC3. C9orf72-/- MEFs show an increase in the ratio of LC3I to LC3II when compared with wild type cells. B) Quantification of the LC3II to LC3I ratio from representative C9orf72-/- and wild-type MEF lines from three independent experiments. C9orf72-/- MEFs show a significant increase in the ratio of LC3II to LC3I when compared with wild-type littermate controls (n = 3, *p<0.05). C) Immunoblot analysis of neurally differentiated cells derived from C9orf72 KO and wild-type embryonic stem cells. C9orf72-/- cells with enriched motor neurons show a dramatic increase in LC3I compared with wild-type controls. Student’s t test is used and data is presented as mean ± SEM. (TIF) [file pgen.1006443.s002.tif]

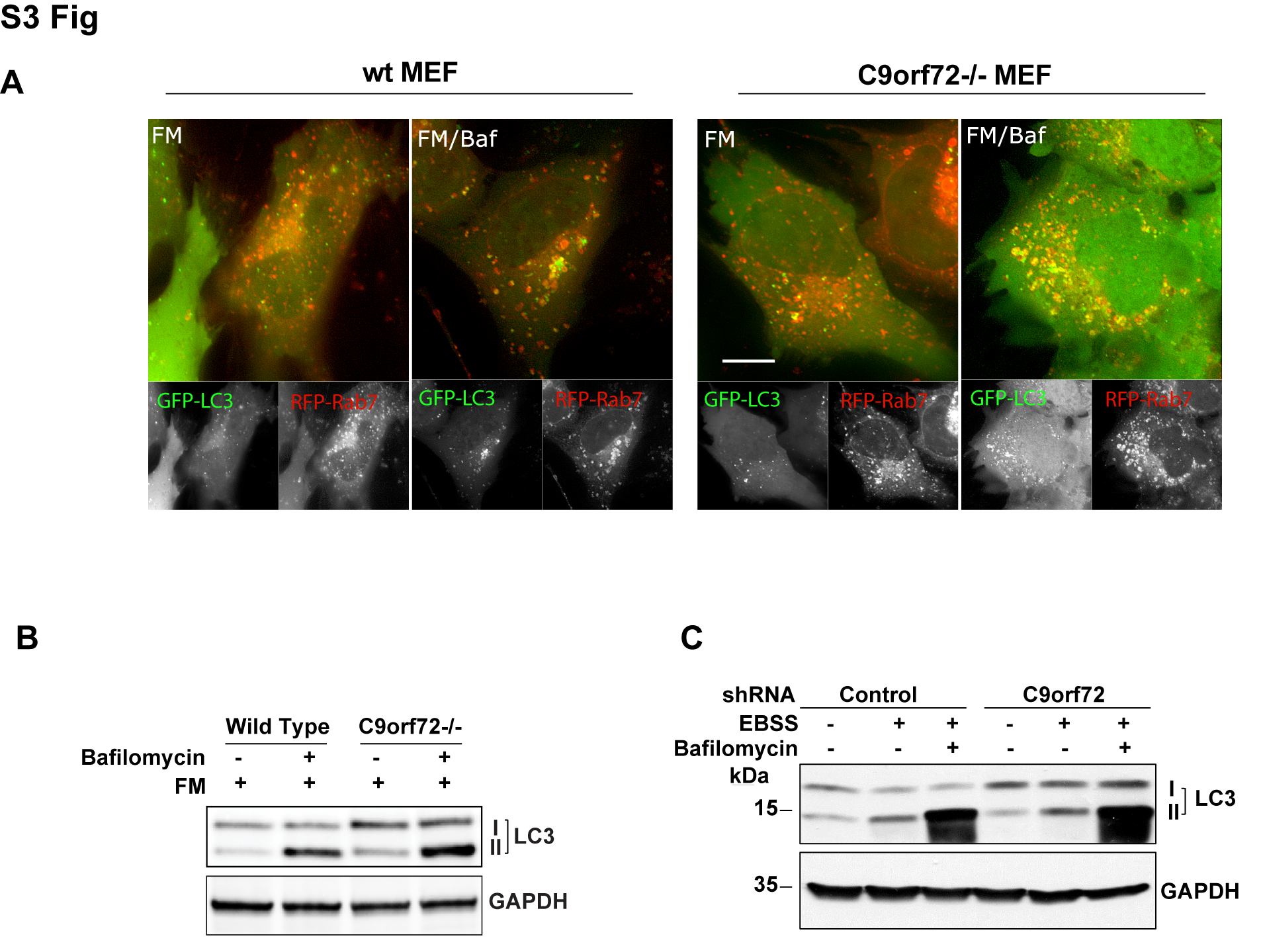

Supplement: S3 Fig — A) Representative live cell images of RFP-Rab7/GFP-LC3 co-localization in C9orf72-/- MEFs. RFP-Rab7 and GFP-LC3 were transfected in wild-type or C9orf72-/- cells and treated with Bafilomycin in fully supplemented medium (FM) conditions. B) Representative image of western blot analysis of LC3 in fully supplemented medium conditions. C) Immunoblot analysis of LC3 levels before and after autophagy induction with nutrient starvation. HEK293T cells were transfected with C9orf72 shRNA or scrambled shRNA control. Approximately 72 hours after transfection, cells were treated with starvation medium (EBSS) with or without Bafilomycin for 2 hours and the resulting lysates were analyzed via immunoblotting. Scale bars: 10 μm. (TIF) [file pgen.1006443.s003.tif]

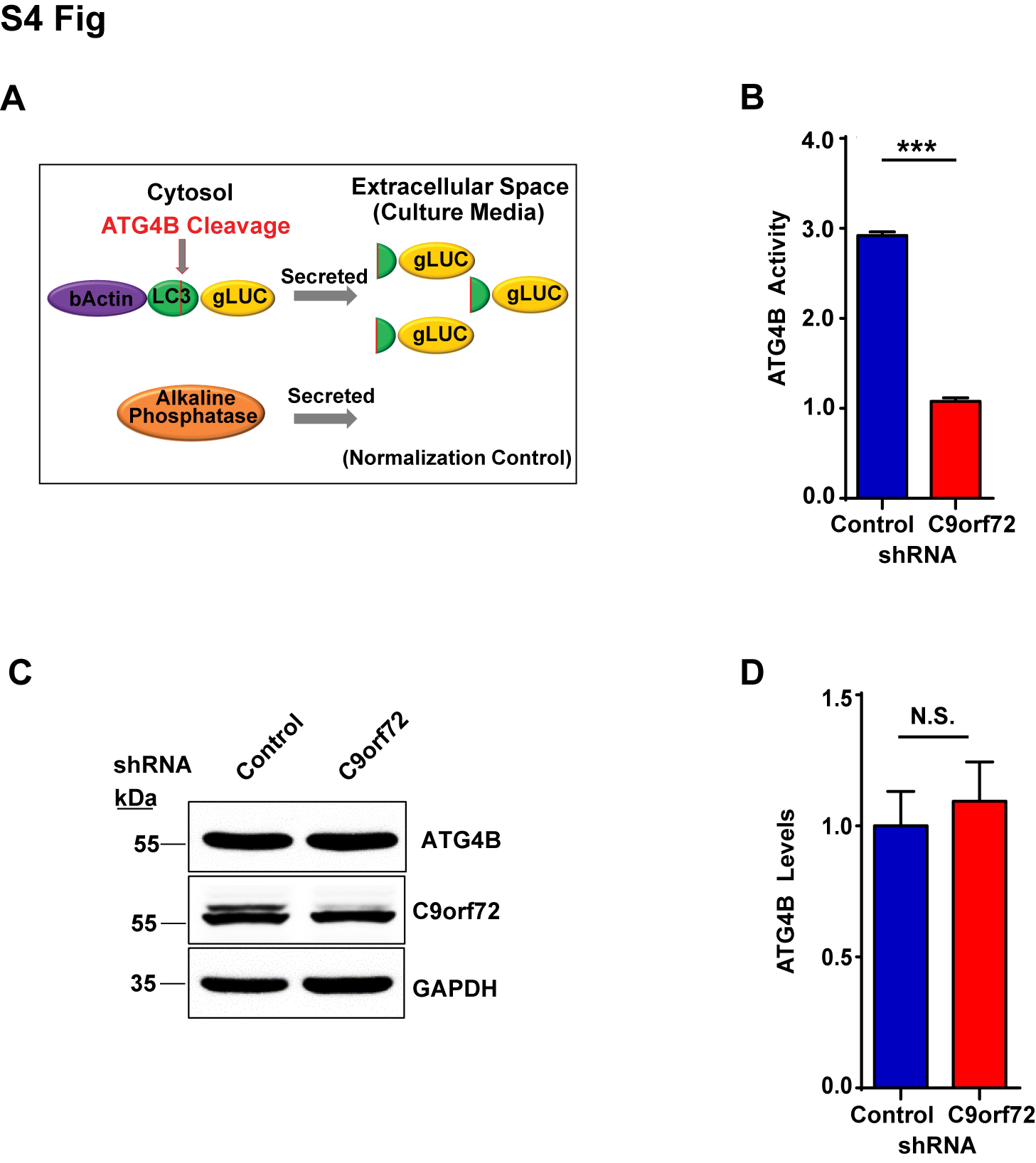

Supplement: S4 Fig — A) Diagram depicting the luciferase release assay. This assay monitors the cleavage of LC3 by the autophagy-associated protease ATG4B. An actin-LC3-GLuc fusion protein, which is fused to the cytoskeleton, is expressed along with a constitutively secreted alkaline phosphatase (SEAP) normalization control. The cleavage of LC3 by ATG4B allows for the rapid secretion of the GLuc luciferase into the cell medium where it can be measured using standard luciferase assay methods. B) Quantification of ATG4B activity after shRNA-mediated knockdown of C9orf72. HEK293T cells were co-transfected with scrambled or C9orf72 shRNA, an ATG4B activity luciferase reporter, and a normalization control construct. Graph represents the cumulative luciferase signal from time points taken at 48 and 72 hours post-transfection from three independent experiments. Knockdown of C9orf72 significantly decreased secreted luciferase signal which corresponds to a decrease in ATG4B activity (n = 3, ***p<0.0005). C) Analysis of ATG4B protein levels after shRNA-mediated knockdown of C9orf72 in HEK293T cells. HEK293T cells were transfected with C9orf72 or control shRNA and lysates collected 72 hours after transfection and the indicated proteins detected by immunoblotting. D) Quantification of ATG4B protein levels after shRNA-mediated knockdown of C9orf72 in HEK293T from three independent experiments. Knockdown of C9orf72 does not significantly change ATG4B levels (n = 3). Student’s t test is used and data is presented as mean ± SEM. (TIF) [file pgen.1006443.s004.tif]

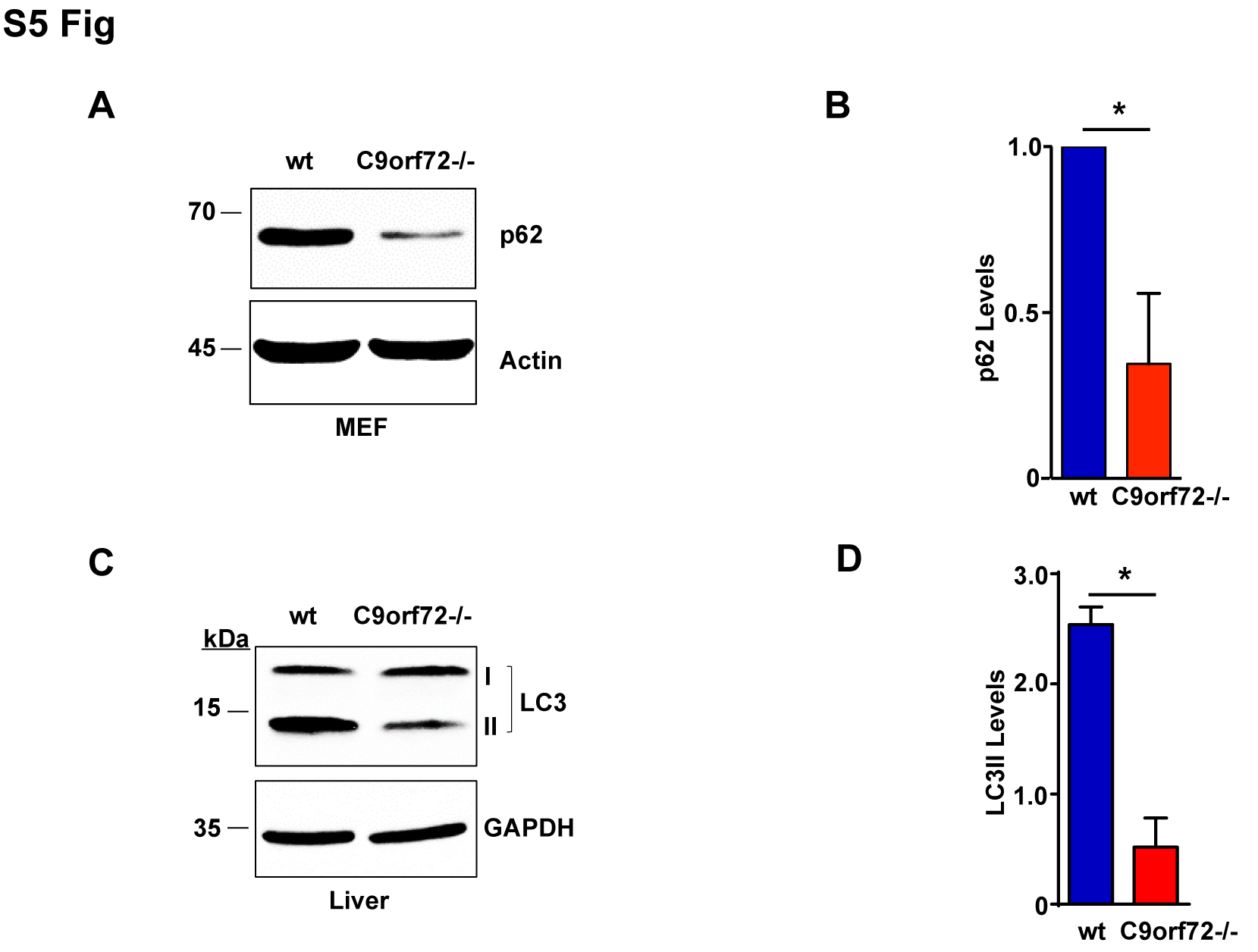

Supplement: S5 Fig — A) Representative immunoblot of p62 protein levels in C9orf72-/- MEFs and wild-type control cells. B) Quantification of p62 protein levels shows a decrease in the C9orf72-/- MEFs (n = 3, *p<0.05). C) Immunoblot analysis of liver homogenates from C9orf72-/- and wild-type animals fed a low-protein diet. C9orf72 KO mice show a decrease in LC3II compared with wild-type littermates. D) Quantification of LC3II levels in liver homogenates derived from C9orf72 KO and wild-type animals fed a low-protein diet. C9orf72 KO mice shows a significant decrease in LC3II when compared with wild-type littermates (n = 2, *p<0.05). Student’s t test is used and data is presented as mean ± SEM. (TIF) [file pgen.1006443.s005.tif]

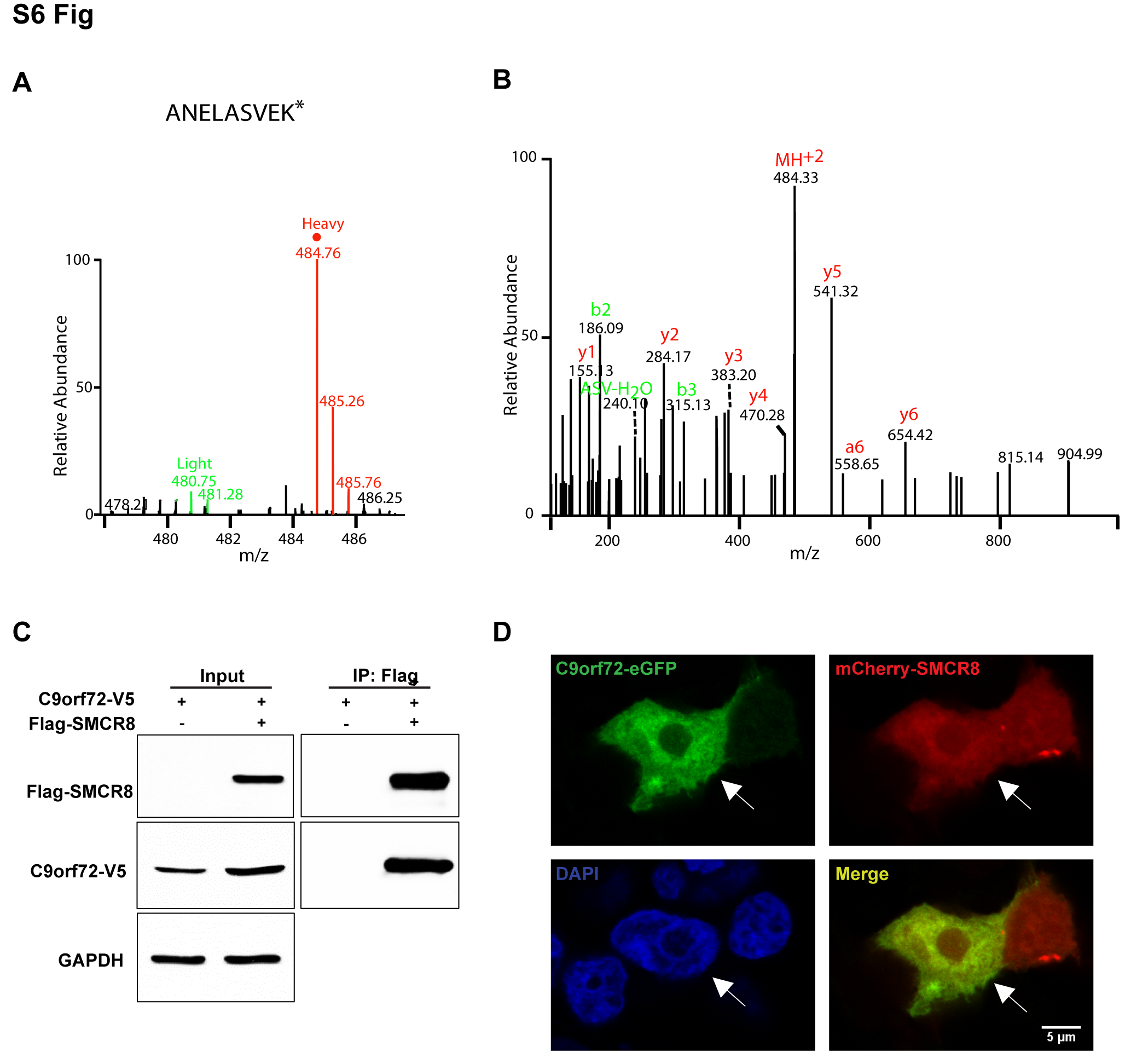

Supplement: S6 Fig — A) Representative MS spectrum of an identified peptide, ANELASVEK for SMRC8. The full MS spectrum of light and heavy forms of the peptide m/z 480.75 and m/z 484.76 and their relative intensity was shown in the spectrum. B) The MS/MS spectrum of the identified spectrum shown in panel (A). C) Validation of the C9orf72-SMCR8 interaction via co-expression in HEK293T cells. HEK293T cells were either co-transfected with Flag-tagged SMCR8 and V5-tagged C9orf72 or transfected with C9orf72-V5 alone and the resulting lysates were incubated with anti-Flag-conjugated beads. V5-tagged C9orf72 successfully co-immunoprecipitates with Flag-SMCR8 and does not bind to beads alone. D) Colocalization of C9orf72 and SMCR8 in HEK293T cells. Cells were transfected with GFP-tagged C9orf72 and mCherry-tagged SMCR8 and imaged using confocal microscopy. Arrow points to a cell expressing both constructs. (TIF) [file pgen.1006443.s006.tif]

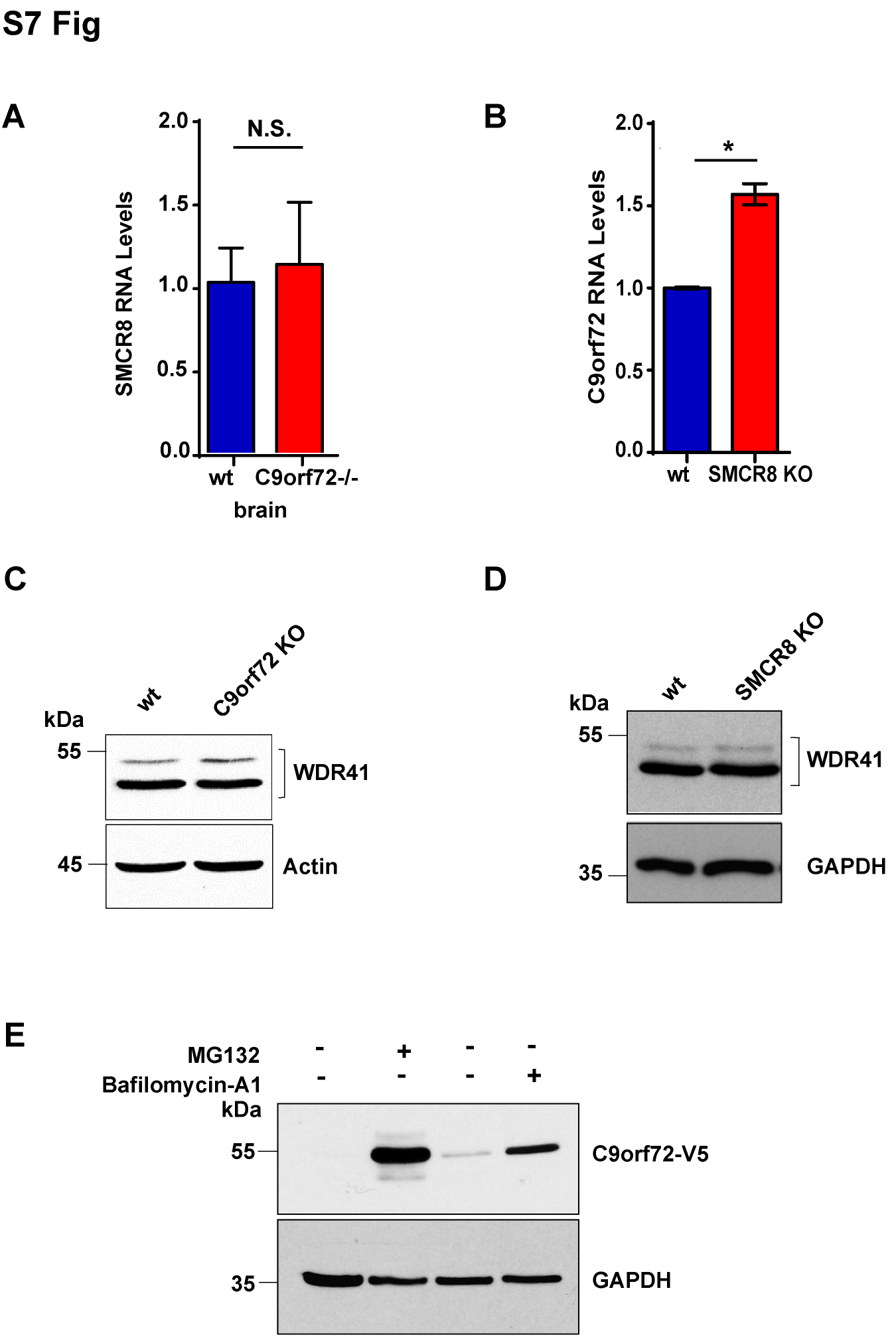

Supplement: S7 Fig — A) qPCR analysis of SMCR8 RNA levels in mouse brain. RNA was isolated from wild-type or C9orf72 KO littermates and SMCR8 transcript levels were assessed by qPCR. No significant change in transcript levels were observed between samples (n = 2) B) qPCR analysis of C9orf72 RNA levels in HAP1 SMCR8 knockout cells. RNA was isolated from control or SMCR8 KO cells and C9orf72 transcript levels assessed by qPCR. No decrease in transcript levels was observed in SMCR8 KO cells as compared to control (n = 2, *p<0.05). C) Immunoblot analysis of C9orf72-/- mouse and wild-type littermate brain homogenates shows no change in WDR41 protein levels in C9orf72 KO animals compared with wild-type control animals. D) Immunoblot analysis of control and SMCR8 KO HAP1 cells shows no change in WDR41 protein levels. E) Analysis of C9orf72 protein levels after MG132 or Bafilomycin treatment. HEK293 cells were transfected with C9orf72-V5 and treated with MG132 (5 μM) or Bafilomycin (100 nM) for 16 hours and the indicated proteins analyzed by immunoblotting. C9orf72 protein levels were increased after MG132 or Bafilomycin treatment. Student’s t test is used and data is presented as mean ± SEM. (TIF) [file pgen.1006443.s007.tif]

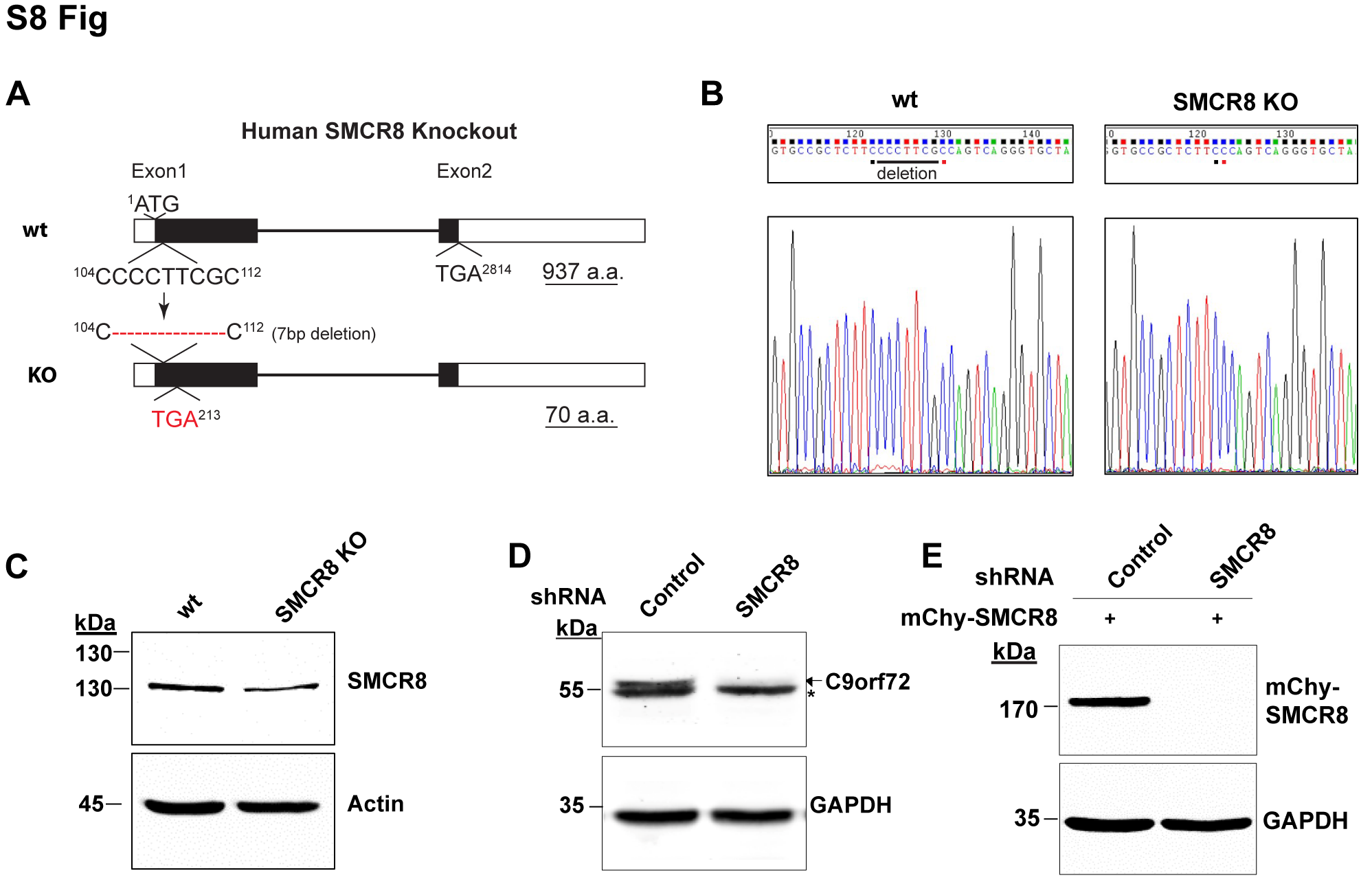

Supplement: S8 Fig — A) Schematic representation of the CRISPR/Cas9 generated SMCR8 KO cell line. HAP1 cells were engineered to contain a 7-base pair deletion in exon 1 of the SMCR8 gene. This mutation is predicted to have an early stop codon and result in the loss of the full-length protein product. B) Validation of SMCR8 deletion. Sequencing results of genomic DNA extracted from control and SMCR8 knockout HAP1 cells confirming the presence of the 7-base pair deletion in exon 1. C) Immunoblot analysis of HAP1 cells. Immunoblot analysis of control and SMCR8 KO HAP1 cells using an antibody against SMCR8. We interpret the band detected in the knockout cells to be a cross-reactive protein product. D) Analysis of C9orf72 levels in SMCR8 knockdown cells. HEK293T cells were transfected with control shRNA or SMCR8-targeted shRNA and the resulting lysates analyzed via immunoblotting. Cells treated with SMCR8 shRNA show a decrease in C9orf72 levels when compared to cells treated with scrambled shRNA. E) Validation of SMCR8 shRNA. HEK293T cells were co-transfected with mCherry-tagged SMCR8 and either control shRNA or SMCR8 shRNA and the resulting lysates were analyzed by immunoblotting. SMCR8-targeted shRNA successfully knocked down overexpressed SMCR8, validating our shRNA. (TIF) [file pgen.1006443.s008.tif]
