# Supplementary material for: Loss of C9orf72 Enhances Autophagic Activity via Deregulated mTOR and TFEB Signaling
Source: PLoS Genet. 2016 Nov 22;12(11):e1006443. doi: 10.1371/journal.pgen.1006443 (PMC5119725; doi:10.1371/journal.pgen.1006443)
Supplement: S1 Table — The top hits with the heavy/light SILAC ratio more than five are shown. (PDF) [file pgen.1006443.s009.pdf]

Gene symbol	Protein description	Refseq_Protein accession	Σ# Unique Peptides	Σ# PSMs	C9orf72/control: Heavy/Light
**C9orf72**	protein C9orf72 isoform a	NP_060795.1	15	355	**14.1**
**SMCR8**	Smith-Magenis syndrome chromosomal region candidate gene	NP_658988.2	3	5	**11.2**
**SLC25A11**	mitochondrial 2-oxoglutarate/malate carrier protein isoform 3	NP_001158890.1	1	15	**6.5**
**TUBB2B**	tubulin beta-2B chain	NP_821080.1	1	150	**6.4**
**WDR41**	WD repeat-containing protein 41	NP_060738.2	5	9	**5.8**
**FAF2**	FAS-associated factor 2	NP_055428.1	4	7	**5.8**
**SLC25A5**	ADP/ATP translocase 2	NP_001143.2	2	45	**5.7**
**DNAJA2**	dnaJ homolog subfamily A member 2	NP_005871.1	4	11	**5.5**
**DNAJC11**	dnaJ homolog subfamily C member 11	NP_060668.2	3	7	**5.5**
**SSR1**	translocon-associated protein subunit alpha precursor	NP_003135.2	2	6	**5.4**
**PCBP2**	poly(rC)-binding protein 2 isoform g	NP_001122386.1	1	6	**5.2**
**SLC25A6**	ADP/ATP translocase 3	NP_001627.2	3	38	**5.0**
**AFG3L2**	AFG3-like protein 2	NP_006787.2	3	5	**5.0**

S1 Table
