# Supplementary material for: Loss of C9orf72 Enhances Autophagic Activity via Deregulated mTOR and TFEB Signaling
Source: PLoS Genet. 2016 Nov 22;12(11):e1006443. doi: 10.1371/journal.pgen.1006443 (PMC5119725; doi:10.1371/journal.pgen.1006443)
Supplement: S2 Table — The DNA primer sequences used for quantitative PCR are provided. (PDF) [file pgen.1006443.s010.pdf]

	qPCR Primers			
**Gene**	**Species**	**Forward**	**Reverse**	**Source**
SMCR8	Mouse	AGCTTATTGGCCTACAAAGAGTGG	GTATAACGGCTGTAACGGCTCAG	PrimerBank
C9orf72	Human	GGGCTCCAAAGACAGAACAG	ATCCCCATTCCAGTTTCCAT	n/a
GAPDH	Human	AAGGTGAAGGTCGGAGTCAAC	GGGGTCATTGATGGCAACAATA	n/a
Actin	Mouse	GGCTGTATTCCCCTCCATCG	CCAGTTGGTAACAATGCCATGT	n/a

S2 Table
